# Estimating RNA-quality using GeneChip microarrays

**DOI:** 10.1186/1471-2164-13-186

**Published:** 2012-05-14

**Authors:** Mario Fasold, Hans Binder

**Affiliations:** 1 Interdisciplinary Center for Bioinformatics, Universität Leipzig, Haertelstr 16-18, Leipzig, D-4107, Germany; 2 LIFE - Leipzig Research Center for Civilization Diseases, Universität Leipzig, Leipzig, Germany

## Abstract

**Background:**

Microarrays are a powerful tool for transcriptome analysis. Best results are obtained using high-quality RNA samples for preparation and hybridization. Issues with RNA integrity can lead to low data quality and failure of the microarray experiment.

**Results:**

Microarray intensity data contains information to estimate the RNA quality of the sample. We here study the interplay of the characteristics of RNA surface hybridization with the effects of partly truncated transcripts on probe intensity. The 3′/5′ intensity gradient, the basis of microarray RNA quality measures, is shown to depend on the degree of competitive binding of specific and of non-specific targets to a particular probe, on the degree of saturation of the probes with bound transcripts and on the distance of the probe from the 3′-end of the transcript. Increasing degrees of non-specific hybridization or of saturation reduce the 3′/5′ intensity gradient and if not taken into account, this leads to biased results in common quality measures for GeneChip arrays such as affyslope or the control probe intensity ratio. We also found that short probe sets near the 3′-end of the transcripts are prone to non-specific hybridization presumable because of inaccurate positional assignment and the existence of transcript isoforms with variable 3′ UTRs. Poor RNA quality is associated with a decreased amount of RNA material hybridized on the array paralleled by a decreased total signal level. Additionally, it causes a gene-specific loss of signal due to the positional bias of transcript abundance which requires an individual, gene-specific correction. We propose a new RNA quality measure that considers the hybridization mode. Graphical characteristics are introduced allowing assessment of RNA quality of each single array (‘tongs plot’ and ‘degradation hook’). Furthermore, we suggest a method to correct for effects of RNA degradation on microarray intensities.

**Conclusions:**

The presented RNA degradation measure has best correlation with the independent RNA integrity measure RIN, and therefore presents itself as a valuable tool for quality control and even for the study of RNA degradation. When RNA degradation effects are detected in microarray experiments, a correction of the induced bias in probe intensities is advised.

## Background

Measurement of gene expression is based on the assumption that an analyzed RNA sample closely represents the amount of transcripts in vivo. Several effects can distort the abundance of RNA transcripts during extraction and preparation before RNA analytics using, e.g., microarrays: The first problem concerns the degradation of the RNA in vitro [1-4]: The quality of purified RNA is variable and after the extraction during storage rather unstable (see [5] and references cited therein). Especially long mRNA fragments up to 10 kb are very sensitive to degradation through cleavage of RNAses introduced by handling with RNA samples. Moreover, transcripts show stability differences of up to two orders of magnitude in vivo, raising the possibility that partial degradation during cell lysis could cause a variable extent of bias in quantification of different transcripts [6]. The second problem concerns amplification of RNA in samples analyzed on microarrays giving rise to the decrease in the length of products that are reverse transcribed and amplified using T7 polymerase [7,8]. The multiple rounds of in vitro transcription that are used to generate samples from small amounts of RNA thus induce a decrease in transcript yield and length.

The screening of nearly three thousand public available GeneChip array data suggests that there is noticeable degradation effect in the majority data files and that 2% of the files were even so severely degraded that their worth was questionable [9]. Working with low-quality RNA may strongly compromise the experimental results and lead to erroneous biological conclusions. It is therefore recommended that the highest quality RNA be used for analyses. However, in some cases, such as human autopsy samples or paraffin embedded tissues, high quality RNA samples may not be available [10-12]. It is therefore important to understand how RNA quality affects the interpretation of the results and also how reliable current quality measures are at indicating RNA quality issues. The assessment of RNA integrity is a critical first step in obtaining meaningful gene expression data. A second step comprises developing methods to quantify degradation and, most importantly, to correct the induced degradation bias in the data and thereby provide more coherent expression measures.

Several RNA quality measures are established based on conventional wet lab techniques such as gel optical density measurement or denaturating agarose gel-electrophoresis (see refs. [2,5] for a review). More novel lab-on-chip gel electrophoresis techniques like Agilents Bioanalyzer became now state of the art. In combination with sophisticated analysis algorithms processing the shape of the electropherogram (and, particularly, the 28 S/18 S rRNA ratio) they provide accepted integrity measures such as the DegFac-RQS (degradation factor RNA quality scale) [6] or the RIN (RNA integrity number) [13] which have been validated independently using qRT-PCR [5].

Importantly, microarray intensity data itself contains information about the RNA quality used for hybridization due to the 3′/5′-gradient of transcript abundance [14]. On microarrays of the GeneChip-type this gradient is typically measured using either specially-designed control probes or exploiting the specifics of the Affymetrix probe design which is based on a set of about one dozen, surface-attached 25-mers interrogating different positions along each transcript. Both options estimate transcript abundance at close and more distant positions towards the 3′-end based on the hybridization signal [15,16].

Although proven in many applications, these measures are based on probe intensities which, in general, are non-linear functions of transcript abundance [17-20]. The signals can be strongly distorted by effects not related to transcript concentration such as saturation and non-specific background hybridization. Intensity-based RNA quality measures are therefore potentially prone to systematic errors which, in worst case, can provide diametrically opposed information in assessing apparently good RNA quality in samples with largely degraded RNA (see below). Moreover, the important task of correcting microarray signals for RNA degradation effects remained unsolved at least in single chip applications to our best knowledge. A linear correction model requiring both expression and RNA quality data from a series of arrays has recently been published [4].

This publication addresses the following tasks to overcome these problems: Firstly, we adapt non-linear hybridization theory based on a physico-chemical model of probe/target binding to the special case of truncated transcripts due to RNA degradation. We will show that our approach consistently explains previous observations such as the effect of RNA quality on transcript intensity level [4] and correlations between probe intensity and probe position along the transcripts and their effect on expression measures [21]. Analysis of the probe signals in terms of this model enables us to define unbiased (in the frame of the hybridization model used) measures of RNA integrity. Secondly, we compare these new measures with established ones. We demonstrate that methods such as *affyslope* or the RNA-integrity control probes can provide systematically false information on RNA quality. Thirdly, we propose a simple correction method which aims at removing the degradation bias from the probe intensities and which can be integrated into standard preprocessing pipelines. Details of the methods used are summarized in the Methods section at the end of the paper or shifted into the supplementary text provided as Additional file 1.

## Results and discussion

### 3′-biased transcript coverage of microarray probes after RNA amplification and degradation

Affymetrix expression microarrays typically use a 3′-biased probe location which is motivated by the specifics of target preparation prior to hybridization. The preparation step applies in vitro transcription (IVT) protocols according to the Eberwein method [22]. It starts with first-strand cDNA synthesis from source mRNA using T7 oligo(dA) primers followed by second strand cDNA synthesis [7,8]. The double-stranded cDNA fragments are subsequently transcribed into amplified antisense RNA (aRNA) which, after labeling, is finally hybridized on the arrays.

**Figure 1:**
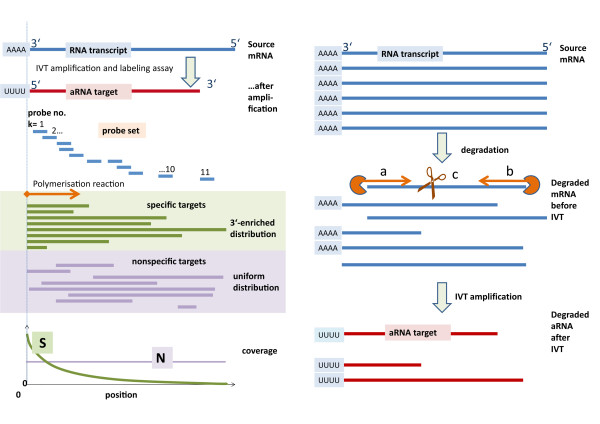
**The 3′-bias of transcript abundance can be caused by in vitro transcription (left part) and degradation (right part) of source mRNA.** Left part: Specific targets hybridize to the probes along the interrogated transcript with decreasing frequency due to incomplete amplification starting at the primers attached to the 3′-poly-A motif of source mRNA. In contrast, cross-hybridization of non-specific targets is not associated with the 3′-end of the transcripts giving rise to uniform coverage. Right part: Degradation of source mRNA due to RNases from both ends **(a** and **b)** and/or fragmentation at randomly chosen positions **(c)** also result in a 3′-enriched length distribution of amplified RNA giving rise to a similar coverage of the probes as shown in the left part. aRNA fragments are shown in 3′ → 5′ direction (from left to right) in contrast to convention to agree with the probe numbering used (k = 1, 2…) and the intensity decays introduced below

First-strand cDNA polymerization is primed at the 3′-end of mRNA and proceeds towards the 5′-end (see Figure 1). Due to incomplete polymerization this method produces truncated transcripts of variable length which are however characterized by a common 3′-start site with respect to the respective fragment of source mRNA [14] (to avoid confusion we will strictly refer to the 3′- and 5′-ends of the source mRNA and not to that of the product aRNA). In consequence, the resulting distribution of transcript lengths gives rise to a 3′-enriched, decaying towards the 5′-end coverage of the probes of the probe sets interrogating the respective transcript with increasing probe index (see also the next section; for convenience we will count the probes in direction towards the 5′-end in contrast to Affymetrix counting the probes in the opposite direction). Subsequent fragmentation of these aRNA targets into pieces of typically a few hundred bases before hybridization leaves the 3′-bias of probe coverage unaffected.

Importantly, the decaying coverage of the probes is expected to apply to specific (S) but not to non-specific (N) hybridization. In the S-hybridization mode the probes bind the aRNA fragments of complementary sequence transcribed from mRNA transcripts which they intend to detect. In the N-hybridization mode the probes bind aRNA fragments of partly complementary sequence originating however from mRNA transcripts not referring to the interrogated gene. Trivially, these non-specific transcripts lack a common start position with respect to the intended target and, as a consequence, they, on the average, uniformly cover the probes of each probe set (see Figure 1a). Specific hybridization competes with non-specific one. Both hybridization modes contribute to the measured probe intensities. The consequences of different probe coverages for the measured signal will be discussed below.

Also degradation of mRNA, e.g. upon storage, can produce 3′-biased probe coverages of fragmented aRNA by endonuclease activity that cuts RNA internally, or by means of exonucleases [23]. In the first case, the poly(A) tail is removed by a deadenylase activity, followed by two mechanisms that degrade the mRNA: either decapping followed by a 5′-to-3′ decay or a 3′-to-5′ decay. Once the mRNA poly(A) tail is removed, reverse transcription reaction will not proceed, resulting in low concentrations of truncated transcripts (see Figure 1b). Several studies have identified RNA degradation to be a major cause of microarray expression measure variability [4,6,9-12].

### Probing transcript abundance using genechip arrays

 With only 25 base-pairs, Affymetrix has considerably shorter probe sequence lengths than competing manufacturers of high-density microarrays such as Illumina or Agilent, who employ probe lengths between 50 and 80 bp. Small probe lengths reduce the specificity for probe/target binding with negative consequences for the signal to noise ratio of the probe intensities. Affymetrix compensates the decrease in single probe signal quality by targeting each transcript with several probes forming a so-called probe set. We here focus on Affymetrix 3′-expression arrays which use typically 11 to 16 probes per set to interrogate transcripts preferentially near their 3′-end (see Figure 1 for illustration).

**Figure 2:**
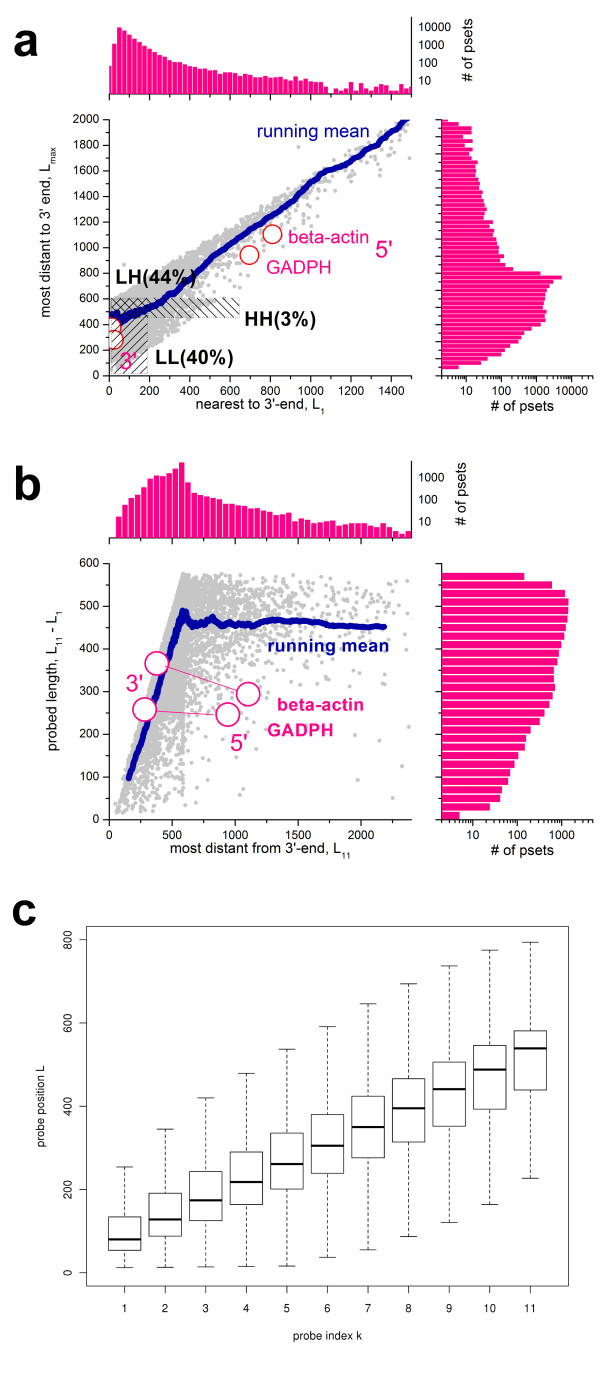
**Probe and probe set characteristics of the RAE230 GeneChip array: Panel a correlates the position of the 11**^**th **^**(nearest the 5′ end of the transcripts) and of the 1th (nearest the 3′ end) probe of each probe set and shows the respective number distributions.** Most probe sets accumulate in the LH (low L_1_, high L_11_) and LL ranges whereas only a few sets are found in the HH range. Panel **b** shows the coverage size of the probe sets (ΔL = L_11_- L_1_) as a function of the position of the 11^th^ probe set together with the respective number distributions. The mean ΔL value nearly linearly increases until k_11_ ≈ 600 and then it remains virtually constant with ΔL ≈ 460. The most probe sets cover a transcript range of 400 – 550 nucleotides. The open circles refer to the 3′- and 5′-control probe sets. The boxplot in part c correlates the probe index k with the probe position L. The median position per index (see the horizontal bar in each box) nearly linearly increases with k. The slope provides the < ΔL > −value of the array which characterizes the probe sensitivity per index increment (~ 50 nucleotide positions per index increment)

 The probes of each set cover transcript lengths which largely exceed the length of the individual probes. This design is well suited to study length-dependent alterations of transcript abundance due to RNA degradation and imperfect amplification. Figure 2a shows that the majority of probe sets start (first probe with index k = 1) within the first L_1_ = 100-200 nucleotides nearest to their 3′-end and end at position L_11_ = 250-600 for the last probe (index k = 11). Only about 5% of all probe sets are located beyond the range of 600 nucleotides. Within this range, the sets can be roughly classified into ‘low (i.e., more 3′) L_1_ and low L_11_’ (LL), ‘low L_1_ and high (i.e., more 5′) L_11_’ (LH) and ‘high L_1_ and high L_11_’ (HH) sets where low refers to distances close to the 3′ end and high refers to distances farther towards the 5′ end (see Figure 2a). The mean length of the covered transcript range (ΔL = L_11_ – L_1_) nearly linearly increases with the position of the 11^th^ probe up to L_11_ ≈ 600, and then it remains virtually constant ΔL ≈ 460 (Figure 2b). Hence, short probe sets with ΔL < 300 accumulate near the 3′ end of the transcripts whereas more distant probe sets typically cover a wider length range of the transcripts (350 < ΔL < 600).

 The mean position of all probes on the array with a given index k = 1…11 linearly correlates with k to a good approximation (Figure 2c). The obtained slope characterizes the mean distance between two neighbored probes. It can be interpreted as the probe sensitivity per index increment and depends on the probe design of the particular array type,

(1) $$〈\text{ΔL}〉=\frac{{〈L〉}_{\text{array}}-{〈L_{1}〉}_{\text{array}}}{{〈k〉}_{\text{array}}-1}=\frac{{〈L〉}_{\text{array}}}{{〈k〉}_{\text{array}}}$$

<… > _array_ denotes averaging over all probes of the array. The approximation in the right part assumes a vanishing intercept in good agreement with the data (see Figure 2c). Additional file 1 provides an overview over selected probe design characteristics of different GeneChip types. It shows that the mean position of the first and of the last probe in the probe sets can strongly vary between the different chip types giving rise to a wide range of < ΔL > −values which can change between about 25 and about 60 nucleotides per index increment. These differences refer in first instance to arrays of older and newer generations (e.g., the human genome HGU95a and HG133a arrays and the mouse genome MG74a and MOE430a arrays, respectively). On the other hand, the average span covered by the probe sets is relatively constant for all chip types considered.

 Affymetrix GeneChip arrays include a small number of control probe sets designed to estimate the RNA quality in terms of the 3′/5′ bias. They target the 3′ end, the 5′-end and the middle (m) of relatively long transcripts coding, e.g., beta-actin and glyceraldehyde-3-phosphate dehydrogenase (GADPH) using 20 probes per set. Figure 2a and b shows that the 3′- and the 5′ probe sets of the controls together cover the range of about 700 nucleotides between L_20_ = 281 and L_20_ = 378 for the 3′-probe sets and L_20_ = 942 and L_20_ = 1104 for 5′-probesets of GADPD and beta-actin, respectively.

### Intensity-based degradation metrics

 In this section we discuss the consequences of the 3′-enriched probe coverage on the observed probe intensities. In the following we will subsume the 3′-bias of probe coverage as ‘degradation effect’ independent of its origin (IVT amplification or degradation) for the sake of convenience. Let us first define the probe-specific and the mean degradation ratio averaged over all probes of the array,

(2) $$d_{\text{g,k}}\equiv \frac{\left[S_{\text{g,k}}\right]}{{\left[S_{g}\right]}_{\text{target}}}\;\text{and d=}{〈d_{\text{g,k}}〉}_{\text{all probes}\;,\;\text{all genes}}$$

respectively, which characterize the decrease of the transcript concentration due to the degradation effect. [S_g_]_target_ is the (true) expression degree of a selected gene g given as the total concentration of the target transcripts in the hybridization solution independent of their length. It refers to the target concentration in the absence of degradation and presumes that RNA processing proceeds without 3′/5′ bias. Contrarily, [S_g,k_] denotes the apparent expression degree reported by probe with index k = 1,…,N_pset_ designed to interrogate target g. It is given as the concentration of the RNA fragments which specifically bind to this probe. It consequently refers to the probe coverage which decays with increasing distance of the probe to the 3′-end of the target. Angular brackets < … > _all probes_ denote averaging over all probes of the array. One expects [S]_g,k_ ≤ [S_g_]_target_ and thus d_g,k_ ≤ 1 owing to the 3′-enrichment after incomplete amplification and degradation of the fragments. The probe specific degradation index, d_g,k_, thus characterizes the loss of mRNA material at a given probe position along the transcribed region of the gene. The mean degradation index d averages the single probe effects over all probes. It estimates the total loss of RNA probed by the microarray in a given preparation prior to hybridization.

 The probe intensity measured in the microarray experiment is given to a good approximation by the hyperbolic function [18,19,24-27]

(3) $$\begin{array}{ll} I_{p}^{P}= & \text{Mx}\frac{X_{p}^{\text{P,S}}\times w_{p}^{\text{P,S}}+X_{p}^{\text{P,N}}\times w_{p}^{\text{P,N}}}{1+\left(X_{p}^{\text{P,S}}+X_{p}^{\text{P,N}}\right)}+O\\ & \text{with}\;p\equiv \text{g,k} \end{array}$$

where M is the maximum intensity upon complete saturation of the probes and O is the optical background. The probe index p ≡ g,k subsumes the gene and probe index explicitly used in Eq. (2). The superscript P = PM, MM specifies the probe type and h = S, N the hybridization mode (specific and non-specific, see below). w_p_^P,h^ is the survival factor characterizing the removal of probe-bound targets in the post-hybridization washing step [26,28,29]. The binding strengths of a selected probe due to specific and non-specific hybridization are directly related to the concentrations of the respective RNA species present in the hybridization solution

(4) $$\begin{array}{l} X_{p}^{\text{ρ,S}}=\left[S_{p}\right]\times {Κ}_{p}^{\text{P,S}}=d_{p}\times {\left[S_{g}\right]}_{\text{target}}{\text{×Κ}}_{p}^{\text{P,S}}\\ \text{and}\;X_{p}^{\text{P,N}}\;=d\times {\left[N\right]}_{\text{chip}}{\text{×Κ}}_{p}^{\text{P,N}} \end{array}$$

respectively. K_p_^P,S^ and K_p_^P,N^ are the respective equilibrium constants for target/probe binding. The degradation factors in Eq. (4) consider the reduction of the concentrations [S_g__target_ and [N]_chip_ after incomplete amplification and/or degradation (see Eq. (2)). Non-specific hybridization is related to the total amount of RNA used for hybridization [30]. [N]_chip_ is consequently reduced by a factor given by the mean degradation factor d.

 The probe-specific degradation index d_p_ defines the decrease of transcript concentration after amplification and degradation (Eq. (2)). In the next step we define the apparent degradation index as the intensity ratio of probes located at different position along the target sequence, for example near its 5′- and 3′-end of one selected target,

(5) $$r_{5^{\prime }3^{\prime }}^{\text{app}}\equiv \frac{I_{5^{\prime }}^{P}}{I_{3^{\prime }}^{P}}$$

where the intensities are given by Eqs. (3) and (4) with the respective degradation ratios d_5’_ and d_3’_, respectively.

Let us consider two special cases if the probes hybridize either far from saturation in the linear range $\left(X_{p}^{\text{P,S}}{\text{,X}}_{p}^{\text{P,N}}\text{<<}1\right)$ or in the range of saturation of specific hybridization $\left(X_{p}^{\text{P,S}}\;{\text{>1>X}}_{p}^{\text{P,N}}\right)$. The apparent degradation index becomes

(6) $$\begin{array}{l} r_{\text{lin}}^{\text{app}}=\;\frac{\left(d_{5^{\prime }}\times X_{5^{\prime }}^{S}{\text{+d×X}}_{5^{\prime }}^{N}\right)}{\left(d_{3^{\prime }}{\text{×X}}_{3^{\prime }}^{S}{\text{+d×X}}_{3^{\prime }}^{N}\right)}=\left\{ \begin{array}{c} r_{5^{\prime }/3^{\prime }}^{\text{true}}\left({Κ}_{5^{\prime }}^{\text{P,S}}{\text{×w}}_{5^{\prime }}^{\text{P,S}}{\text{/Κ}}_{5^{\prime }}^{\text{P,S}}{\text{×w}}_{3^{\prime }}^{\text{P,S}}\right)\;\text{for}\;x^{S}\;\text{>>}\;x^{N}\;\left(\text{specific}\right)\\ \left({Κ}_{5^{\prime }}^{\text{P,N}}{\text{×w}}_{5^{\prime }}^{\text{P,N}}{\text{/Κ}}_{3^{\prime }}^{\text{P,N}}{\text{×w}}_{3^{\prime }}^{\text{P,N}}\right)\;\text{for}\;x^{S}\;{\text{<<x}}^{N}\left(\text{non-specific}\right) \end{array}\left(\text{linear range}\right)\right.\\ \text{and}\;r_{\text{sat}}^{\text{app}}=\frac{w_{5^{\prime }}^{\text{P,S}}}{w_{3^{\prime }}^{\text{P,S}}}\;\left(\text{saturation}\right) \end{array}$$

respectively. The lower case x defines the hybridization strengths at ‘ideal’ transcript concentrations (see Eq. (4) with d = d_p_ = 1: $x_{p}^{\text{P,S}}\equiv {\left[S\right]}_{\text{target}}\times {Κ}_{p}^{\text{P,S}}\times w_{p}^{\text{P,S}}\;\text{and}\;x_{p}^{\text{P,N}}\equiv {\left[N\right]}_{{}_{\text{chip}}}\;{\text{× Κ}}_{p}^{\text{P,N}}\;\text{×}w_{p}^{\text{P,N}})$ and $r_{5'/3'}^{\text{true}}\equiv d_{5'}/d_{3'}$ denotes the ‘true’ relative degradation index between 5′ and 3′ probes, respectively. Eq. (6) shows that the apparent degradation index is proportional to the true one $\left(r_{\text{lin}}^{\text{app}}\propto r_{5'/3'}^{\text{true}}\right)$ in the special situation of dominating specific hybridization (x^S^> > x^N^) far from saturation only. It however scales with the ratio of the specific binding and washing constants of the 3′- and 5′-probes, which might be larger or smaller than unity depending on the sequences of the particular probes (see [29] for details). At dominating non-specific binding or saturation one gets apparent degradation indices which are completely independent of the true one. Their values again depend on the probe sequences and can be larger or smaller than unity. Hence, the use of intensity-based degradation metrics raises problems because they reflect the degradation bias of transcript abundance in special situations only.

On the other hand, two intensity-based degradation measures are well established for quality control of GeneChip arrays: (i) The slope of a linear function fitted to the so-called ‘RNA degradation plot’, r_5’/3’_^slope^. This RNA degradation plot displays the mean logged intensity averaged over all probes with the same index k, taken from one array, as a function of k [16]. (ii) The intensity ratio r_5′/3′_^control^ of special control probe sets targeting the 5′- and the 3′-end of relatively long transcripts such as beta-actin and GADPH. A threshold of the 3′/5′-signal intensity ratio of the GADPH controls less than 3 (in logarithmic scale log_10_ 3 = 0.48) is recommended for good quality RNA [31,32].

In view of the discussed problems of intensity-based degradation measures we will revise these estimates and judge their suitability for determining RNA quality. Large values of r_5′/3′_^slope^ and/or r_5′/3′_^control^ near unity are generally thought to indicate small degradation bias and thus good RNA quality. Note that reciprocal values of these measures are often used in practice estimating the respective 3′/5′-ratios. Here we consequently use 5′/3′-ratios to ensure direct comparability between the various measures.

In summary, probes located nearer to the 3′-end of the interrogated transcripts potentially shine brighter than more distant probes due to the 3′-enrichment of probe coverage giving rise to expected ‘true’ intensity ratios r_5′/3′_ < 1. However, this rule applies only to conditions of specific hybridization far from saturation. RNA quality measures based on the 5′/3′-intensity ratio consequently require consideration and evaluation of the hybridization mode of the chosen probes. Moreover, the potential dependence of the probe intensities on the degree of degradation gives rise to systematic errors of the estimated expression degree of the transcripts which requires appropriate correction.

### The 3′-intensity bias depends on the hybridization-mode

**Figure 3:**
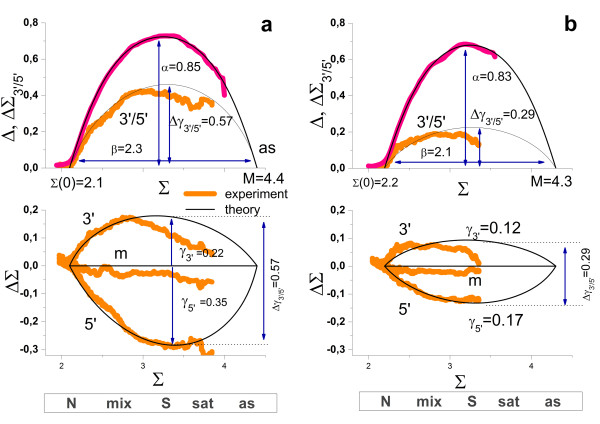
**Hook- and degradation hook (above) and tongs-plot (below) of two selected chip hybridization taken form the human body index data set (muscle, GEO accession numbers GSM176301 in part a and skin, GSM175967 in part b) referring to large and smaller degradation effects, respectively.** Note that all plots use the same abscissa scaling (Σ, see Eq. (7)) which is related to the expression degree of the respective probes. The hook curve reveals the changing hybridization mode with increasing sigma: non-specific (N), mixed N and S (mix), specific (S), saturation (sat) and asymptotic (as) ranges. The degradation hook and the tongs-plot reveal the mean 3′/5′-intensity bias of the probes. The three branches of the tongs plot refer to three probes nearest to the 3′-end (upper branch), nearest to the 5′-end (lower branch) and located in the middle in-between (middle branch). Note that the different branches split maximally in the S-range of hybridization whereas no bias is observed in the N-range as predicted by theory (lines, see Eqs. (16) and (21) for the hook and tongs plot, respectively). The theoretical curves are calculated using the formulae given in the methodical section using the parameters given in the figure. The hook dimensions (α, ‘height’ of the hook, see Eq. (18); β, ‘width’ of the hook; Σ(0), ‘start’ point; M, ‘end’-point) are very similar for both arrays whereas the logarithmic 3′- and 5′-degradation levels (Eq. (24)) are markedly different. The size of the moving window is decreased towards the right end of the tongs plot to compensate the reduced number of probe sets in saturation range. As a consequence, the part of the curves beyond of the maximum is prone to increasing error

The so-called hook method transforms the PM and MM intensities of GeneChip arrays into a smoothed delta-versus-sigma summary plot of characteristic shape where delta and sigma are the difference and the sum of the logged probe intensities after affinity correction. Its visual inspection allows the simple and straightforward detection of five hybridization regimes with increasing sigma (see [33,34], Eq. (14) and Figure 3), namely the *N-* (virtually only non-specific hybridization contributes to the signals), *mix-* (combination of non-specific and specific hybridizations), *S-* (predominantly specific hybridization), *sat*- (saturation range; the relation between intensity and transcript concentration becomes progressively non-linear) and *as-* (the intensity reaches its asymptotic saturation level) regime.

We here complement the hook representation by two modifications allowing to assess the degradation of the RNA-transcripts in a chip-specific fashion. These so-called ‘degradation hook’ and ‘tongs plot’ estimate the 3′-enrichment of the probes and thus their degradation level in dependence on the hybridization mode. They depict differences between the Sigma-values of selected subsets of probes taken from the 3′- and 5′-ends of the probe set,

(7) $$\begin{array}{l} \;\text{Δ}\Sigma {}_{S3^{\prime }/S5^{\prime }}\equiv \;{〈\Sigma {}_{p}〉}_{S3^{\prime }}-{〈\Sigma {}_{p}〉}_{S5^{\prime }}\\ \;\left(\text{degradation hook}\right)\\ \Delta \Sigma {}_{S}\equiv {〈\Sigma {}_{p}〉}_{S}-{〈\Sigma {}_{p}〉}_{\text{pset}}\;\left(\text{tongs plot}\right)\\ \text{with}\;{〈\Sigma {}_{p}〉}_{S}\equiv \frac{1}{3}\overset{\text{i+}2}{\underset{\text{k=i}}{\Sigma }}\Sigma_{k}\\ \text{and}\;\Sigma {}_{k}\equiv \frac{1}{2}\left({\text{logI}}_{k}^{\text{PM}}{\text{+logI}}_{k}^{\text{MM}}\right) \end{array}$$

as a function of sigma, ${〈\Sigma_{p}〉}_{\text{pset}}$. These plots use the same abscissa as the hook curve and they also smooth the noisy data using a running window of 500–1000 probes. The subscript s = s3′, s5′ denotes a subset of three consecutive probes within the probe set of size N_pset_ nearest to (s3′, i = 1) or most distant from (s5′, i = N_pset_-2) the 3′-end of the transcript, or centred about its middle probe (s = m).

Figure 3 shows the hook curves for two selected microarray hybridizations of differently degraded RNA together with the respective degradation hook (panel above) and tongs plot (panel below). The degradation hook shows essentially the same shape as the standard hook. The curves reflect however different effects: The standard hook plots the mean logged intensity difference between paired PM and MM probes. It consequently estimates the intensity penalty of one mismatched base pairing in the respective probe/target-duplexes. Contrarily, the degradation hook judges the logged mean intensity difference between probes located nearer and farther to the 3′end of the transcripts, and thus the 3′/5′-bias of the probe intensities in the probe sets due to the degradation effect.

Interestingly, the different hybridization modes analogously affect the intensity differences in the standard and the degradation hook as well. For example, upon non-specific hybridization both, the PM/MM difference and the 3′-bias essentially disappear because both effects, the MM-penalty and the 3′/5′-bias, require duplexing of the probes with the intended targets. Non-specific binding doesn’t meet this criterion because the binding of non-specific transcripts is indifferent with respect to the mismatched pairing of the middle base of the MM probes and with respect to the degradation bias as well (see also Figure 1 for illustration). Vice versa, both hook-versions show their maximum in the S-range because specific binding is associated with the intended intensity penalty of the MM-probes and of probes located more distant from the 3′-end, respectively.

Note that the two standard hook plots shown in panel a and b of Figure 3 are of virtually equal height owing to the similar MM-penalty (α = 0.83 – 0.85) whereas the respective degradation hooks markedly differ in this respect (Δγ_3′/5′_ = 0.57 and 0.29, respectively) revealing marked differences in the degradation level between both samples. Comparison of the heights of both hook-types shows that strong degradation can affect the probe intensities nearly by the same order of magnitude as one mismatched base pairing.

The tongs plots explicitly estimate the intensity bias at three positions of the probe sets and thus it illustrates the progression of degradation with increasing probe index. The ΔΣ_s_ curves of all three subsets (s = 3′, 5′ and m) degenerate in the N-hybridzation range indicating the absence of the 3′-bias for non-specific binding as discussed above (see also Eq. (6) for x^S^ < <x^N^). In the mix-range the ΔΣ_s_-curves split into three branches which progressively diverge with increasing sigma and thus with increasing contribution of specific hybridization. The ‘opening of the tongs’, i.e. the split between the 3′- and 5′-branches, reaches its maximum in the S-range of hybridization in parallel with the maximum of the hook curve and of the degradation hook. Subsequently, the different branches start to converge as predicted for the range of saturation (see Eq. (6)). Both, the experimental degradation hook and the tongs plot are well described by theoretical curves based on the Langmuir-model of array hybridization (see Materials and Methods section, Eq. (21)). The split parameter Δγ_3′/5′_ characterizes the height of the degradation hook, or equivalently, the ‘tongs opening’ serving as a measure of the maximum vertical difference between the 5′- and the 3′-branches of the tongs, respectively. Δγ_3′/5′_ estimates the 5′-depletion of probe coverage in terms of the logged concentration increment between the targets covering the 5′- and 3′-probes (Eq. (24)). The examples shown in Figure 3a and b refer to relatively strong and weak depletion of targets with 5′/3′-concentration ratios of d_tongs_ = 10^-0.57^ = 0.27 and 10^-0.29^ = 0.51, respectively (see Eq. (24)). This analysis shows that degradation can reduce the transcript concentration to less than one third of the initial transcript abundance.

**Figure 4:**
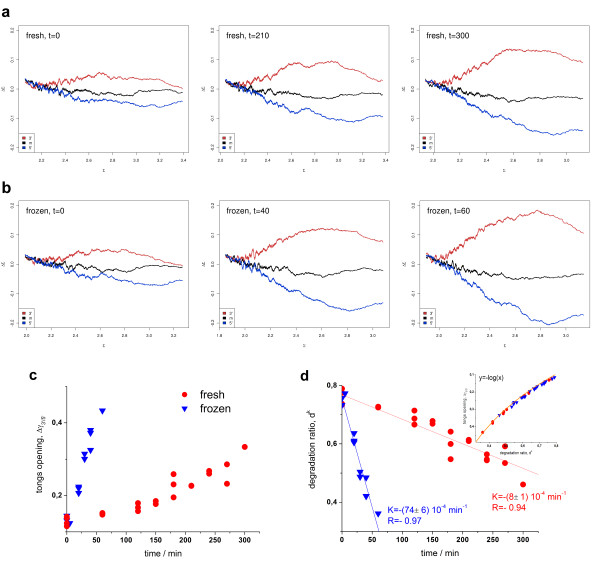
**Collection of tongs plots taken from the ratQC data set.** The RNA was extracted from liver samples either after ex vivo incubation of fresh tissue (panel **a**, incubation time 0, 210 and 300 min) or after thawing frozen tissue (**b**, incubation time 0, 40 and 60 min). The plots in panel **c** and **d** show the tongs-opening and the d^k^ parameter of both series as a function of the incubation time, respectively. RNA prepared from frozen samples degrades much faster than RNA from fresh samples. The insert in part b correlates the d^k^ and tongs opening parameters. Their relation follows a logarithmic function

Figure 4 shows a collection of tongs plots taken from the *RatQC* dataset [10] characterizing the level of degradation of rat liver RNA under two conditions, namely after incubation of fresh tissue (panel a) or after thawing frozen tissue (b). With incubation time the opening of the tongs increases indicating progressive degradation of the RNA. The time dependence reveals that RNA degradation in thawed tissue proceeds much faster: Particularly, its degradation level after 50 min exceeds that of incubated fresh tissue after 300 min in units of the tongs opening parameter, Δγ_3′/5′_ (Figure 4c). It has been argued that freezing disrupts tissue structure, rendering the tissue highly sensitive to RNA degradation whereas autolysis of fresh liver tissue appeared to be a much slower process [10].

In summary, the 3′/5′-bias of probe intensities essentially disappears for probes which hybridize predominantly non-specifically and it markedly decreases for probes which are strongly saturated with specific transcripts. The 3′/5′-bias consequently provides a suited metrics for RNA-quality only in the linear range of specific hybridization in agreement with the theoretical predictions made in the previous subsection.

### Short 3′-probe sets are prone to non-specific′ hybridization

In the next step we selected the probe sets from the non-specific and specific hybridization ranges of the hook curve and calculated their frequency histograms as a function of L_1_ and L_max_, the position of the nearest and of the most distant probe from the 3′-end in each probe set.

**Figure 5:**
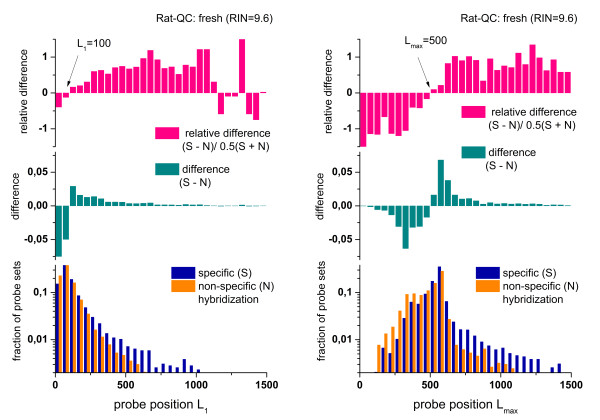
**Distribution of probe sets hybridized predominantly with specific and non-specific transcripts as a function of the position of the first (left part) and last (right part) probe of each probe set.** The graphs show that ‘short’ probe sets located nearer to the 3′-end of the transcript are more prone to bind non-specific transcripts at (L_1_ < 100 and L_max_ < 500) than specific ones. The part in the middle shows the difference between the respective fractions of probe sets whereas the part above normalizes this difference with respect to the mean fraction of probe sets in both, S- and N-groups. Accordingly, the differential binding refers to about 50% of all probe sets. The distributions are calculated using the ratQC data set

Figure 5 shows the distribution of the fraction of probe sets of either hybridization range normalized with respect to the total number of probe sets in the respective group. Probe sets which cover the range near the 3′-end with L_1_ < 100 and L_max_ < 500 are more prone to non-specific hybridization than probe sets located at larger distances from the 3′-end with L_1_ > 100 and L_max_ > 500 which are more affected by specific hybridization on the average. The relative difference of the fractions in both groups is large: For example, the fraction of N-hybridized probe sets exceeds that of S-hybridized ones by about 50% at small L_max_ < 300. Vice versa, at large L_max_ > 700, the S-hybridized fraction considerably exceeds the N-fraction. The observed distributions are very similar for the different arrays of the Rat-QC data set showing that the positional-dependent variation of the hybridization mode is virtually insensitive to the degree of RNA-degradation.

We suspect that the increased fraction of non specific hybridization towards the 3′ end of the transcripts is caused by inaccurate assignment of the 3′-transcript end upon probe design and/or by variations of the 3′-end of the transcripts, e.g. due to effects such as alternative polyadenylation as discussed previously [35,36]. Alternative polyadenylation leads to transcript isoforms with differences in the 3′ UTR length. In these situations the ‘true’ 3′-end of the transcript can be located at L3′ > 0 and all probes at positions closer to the apparent transcript end, L3′ > L > 1, will hybridize exclusively non-specifically owing to the absence of specific transcripts. In consequence, the mean fraction of non-specific hybridization of probes at small L will exceed that of specific hybridization on relative scale, as observed. A very similar plot as shown in Figure 5 for the rat genome array RG230A was obtained for alternative array types such the Human Genome U133 Plus 2.0 (see Additional file 1).

An alternative option that potentially explains the increase of the relative contribution of N-hybridization near the 3′-end can be sought in depletion of the respective targets in solution due to the high number of probes with partly overlapping probe sequences in this L-range. In consequence, a larger number of probes can be thought to compete for each transcript-fragment than at larger distances L. This competition for the same target can deplete its concentration in solution. Such target depletion effectively reduces the binding affinity of the respective probes for specific binding [37]. In consequence this change can increase the relative contribution of non-specific hybridization as observed in this L-range. On the other hand, it has been shown that depletion is clearly governed by the binding affinity of the probes which exponentially affects the abundance of targets whereas the accumulation of partly overlapping probes near the 3′-end can be assumed to affect target concentrations in a linear and thus much weaker fashion. We therefore suspect that target depletion is, if at all, of secondary importance for explaining the high relative contribution of non-specific hybridization at small L.

In summary, we found a biased distribution of specific and non-specific hybridization along the targeted transcripts: Non-specific binding is more heavily weighted near the 3′-end, presumably owing to its inaccurate assignment and to transcript isoforms with variable 3′ UTR lengths.

### Positional dependent intensity decays

The degradation hook and the tongs plot shown in Figure 3 highly resolve the 3′-bias of probe intensities in dependence on the hybridization mode. These plots allow classifying each probe set into one of five different hybridization regimes within a microarray experiment. However, this approach only coarsely resolves the positional bias along the transcripts by collecting together three probe intensity values at two or three selected positions only (3′, 5′ and m). In this subsection we describe an orthogonal method which uses a more coarse graduation of the hybridization mode while highly resolving the 3′-bias with respect to the probe position. Particularly, we select two groups of probe sets taken either from the N- or the S-hybridization range of the hook curve. We then calculated the logged mean intensities of the selected PM-probes as a function of two alternative arguments, namely their probe index k in the probe set or their probe distance L relative to the 3′-end given in units of the number of nucleotides,

(8) $$\begin{array}{l} {\text{logI}}^{h}\left(k\right)=〈{\text{logI}}_{p}^{h}〉|{}_{\text{p=k}}\;\text{and}\\ {\text{logI}}^{h}\left(L\right)=〈{\text{logI}}_{p}^{h}〉|{}_{L_{\text{p=L±δL}}}\text{with h=S,N} \end{array}$$

respectively. The angular brackets denote averaging either over all probes with the same index k or over all probes with the same absolute position within a window L-δL < L_p_ ≤ L + δL.

**Figure 6:**
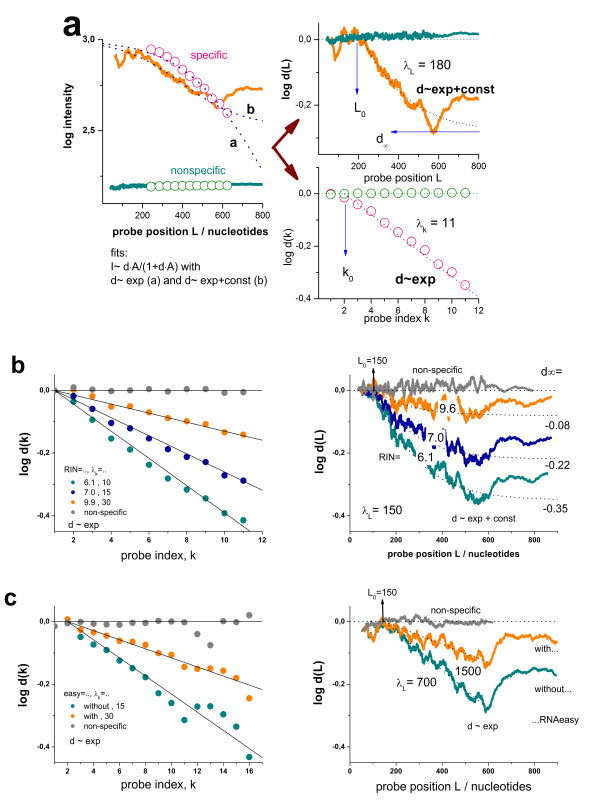
**Positional dependent intensity decays in relative and absolute scale.** Panel **a)** Mean intensity decays of specifically and non-specifically hybridized probes (Eq. (8)) referring to the data shown in Figure 3a. The circles denote index-based averages which are plotted as a function of the mean position per index (left part). The decays are normalized according to Eq. (9) (right part of the figure). The dotted curves in part a are theoretical ones using different functions: Exponential plus constant **(a)** and exponential **(b)** intensity decays which consider saturation without initial shift (Eq. (10) with x_0_=0); exponential plus constant (right part above) and exponential (right part below) decays with initial shifts (Eq. (10) with x_0_=0). Panel **b** and **c**) Representative decays are taken from the Rat-QC **(b)** and the RNeasy cleanup **(c)** data sets. The index-scaled decays in the left part and the L-scaled decay in the right part of panel c are fit using simple exponential decays (d_∞_ = 0) whereas the L-scaled decays in part b in addition use a constant d_∞_ > 0

Figure 6a shows the obtained intensity profiles for the example shown in Figure 3a. The mean intensity due to specific hybridization markedly decays with increasing distance of the probes from the 3′-end of the transcripts whereas the intensity due to non-specific binding is much smaller and remains virtually constant, as expected. The decay due to specific hybridization can be approximated with a distant-dependent degradation index, d_p_^P,S^ = d^S^(L) which is given by an ‘exponential plus constant’ decay law in analogy with Eq. (10) (see below) after insertion into the hyperbolic Langmuir isotherm (see Eqs. (3) -(4)) and normalization (Eq. (9.). The obtained curves well describe the intensity decay in the intermediate L-range and its flattening at small and large L-values (see dotted curve b in Figure 6a).

This approach attributes the flattening of the decay near the 3′-end to the saturation of the probes with bound transcripts. However this effect becomes relevant usually at large intensity values only (log I(3′)~ log M > 4; see Figure 3). The observed mean initial intensity values of the decays are however much smaller (log I(3′) ~ 3). We conclude that another effect and not saturation causes the flattening of the decays at small L-values. The decrease of the relative contribution of specific hybridization near the 3′-end discussed in the previous subsection well explains the observed trend: Non-specific hybridization still adds a small residual contribution to the specific decays due to imperfect decomposition of the different hybridization modes. The decrease of the contribution of specific binding presumably due to inaccurate assignment and transcript isoforms then effectively increases the relative weight of non-specific binding and adds a constant component to the decays at small distances from the 3′end which in consequence flattens the initial decay.

To account for this effect we pursue a simple approach which neglects saturation and normalizes the decays with respect to their maximum intensity level near the 3′ end of the transcripts,

(9) $$\begin{array}{l} d^{h}\left(x\right)=I^{h}\left(x\right){\text{/I}}^{h}\left(3^{\prime }\right)\\ \;\text{with x=k,L and h=N,S} \end{array}$$

The obtained degradation index due to non-specific hybridization is given by a constant, d^N^(x) ≈ 1, to a good approximation (Figure 6). The degradation decays due to specific hybridization are well described using a ‘shifted exponential plus constant’ functions of the form,

(10) $$d\left(x\right)=d^{s}\left(x\right)\approx \left({1-d}_{\infty }^{x}\right)\text{×exp}\left(-\frac{{\text{x-x}}_{0}}{\lambda_{x}}\right){\text{+d}}_{\infty }^{x}$$

as illustrated by the dotted curves in Figure 6. The obtained decay length λ characterizes the mean slope of the 3′-bias in units of the number of probes (λ_k_) or nucleotides (λ_L_) after which the variable contribution of the intensity decays to 1/e of its initial value. The constant d_∞_^x^ defines the residual constant intensity level at large distances from the 3′-end. The shift-parameters x_0_ = k_0_, L_0_ account for the potential flattening of the decay at small arguments discussed above. Both decay constants are linked via the < ΔL > −value, i.e.

(11) $$\lambda_{k}\approx \lambda_{L}\cdot 〈\text{ΔL}〉$$

Panel b and c of Figure 6 show selected examples taken from the rat-QC and the RNeasy cleanup data sets which refer to different array types (RAE 230A and HG-U133A, respectively). With decreasing RNA quality the decays become steeper paralleled by increasing absolute values of the limiting intensity levels but almost constant initial shift parameters L_0_ ≈ 150 – 200 and k_0_ = 1- 2. Index- and nucleotide-based length scales give rise to similar trends (compare the right and the left parts in Figure 6b and c). The L-scale in units of nucleotides is associated with a slightly more flat and smaller asymptotic level than the relative k-scale using the probe indices as argument. Note that about 95% of the probes of the arrays are positioned with similar frequencies in the range 100 < L < 600 whereas only less than 5% of them are found at larger distances, however with a broad distribution over the range 600 < L <2800 (Figure 2). Most of the more distant probes refer to the probe indices k = 10, 11. This assignment effectively compresses the asymptotic region to the last two probes with indices k = 10 and 11. As a consequence the decays in relative k-scale can be described with sufficient accuracy using a ‘single exponential’ decays (Eq. (10) with d_∞_^k^ = 0) where the values d(10) and d(11) roughly refer to the limiting decay level obtained in the fits using the L-scale, d_∞_^L^.

The L-decays of specifically hybridized probes obviously behave differently for L > 600 showing a less pronounced loss of intensity than for L < 600. The origin of this difference is unknown. The standard error of the experimental decays roughly agrees with the symbol size (k-dependencies, left part of Figure 6b and c) or it slightly exceeds line thickness (L-dependencies, right part of Figure 6b and c). The small oscillations in the decays and the relative increase at L > 600 thus reflect systematic effects presumably due to differences of the probe properties in the different subensembles of probes referring to each data point such as their binding affinity and also their degradation degree. Recall that the number of probes drastically decreases at L > 600 which makes this range less relevant for correcting purposes of the majority of probes. We exclude this range therefore from curve fitting.

Our fits show that the values of the decay parameters systematically depend on the chosen decay function and strongly correlate each with another. To illustrate this correlation we show fits with variable d_∞_^L^ but constant λ_L_ = 150 in Figure 6b (right part) and fits with constant d_∞_^L^ = 0 but variable λ_L_ in Figure 6c (right part). The values of the variable parameters d_∞_^L^ and λ_L_ systematically decrease with progressive degradation. Both options equally well describe the decaying part of d(L) in the range 100 < L < 600.

To obtain a robust decay characteristic we substitute the exponential fit functions (Eq. (10)) by a simple two-point estimate

(12) $$\log\;d^{k}={〈{\text{logI}}^{S}〉}_{\text{k=10,}11}-{〈{\text{logI}}^{S}〉}_{\text{k=1,}2}$$

This logged degradation ratio characterizes the intensity decay in the index-range k_start_- k_end_ =2-10, or equivalently, in the positional range L_start_-L_end_ = <L > _1,2_ - < L > _10,11_ ~ 150–550 which comprises the majority of more than 95% of all probes. The degradation ratio can be transformed into estimates of the decay length of the exponential decays: $\lambda_{k}\approx 8/\ln d^{k}$and $\lambda_{L}\approx 8\cdot 〈\Delta L〉/\ln d^{k}$.

Please note that the decay function defined in Eq. (10) estimates the fold change of transcript abundance at position x and x_0_, to a good approximation, i.e.

(13) $$d\left(x\right)\approx {\left[S\right]}_{x}/{\left[S\right]}_{x0}$$

The degradation ratio (Eq. (12)) consequently estimates the mean fold change of transcript abundance reported by the probes positioned near the 5′- and 3′-ends of the probed range. It represents an alternative estimate of the tongs opening parameter introduced above, $d^{k}\propto d_{\text{tongs}}=10^{\Delta \gamma_{3'/5'}}$ (Eq. (22)). Figure 4c shows the time course of RNA degradation in the Rat QC experiment using the tongs opening (panel c) and the d^k^ (panel d) parameters. Both measures strongly correlate (see insertion in Figure 4d) and essentially reflect the same degradation behaviour of the samples studied.

In summary, the effect of degradation can be described as a function of the probe position in terms of a ‘shifted exponential decay plus constant’-function using either the probe index or the ‘absolute’ probe position as argument. This information can be further condensed into a single degradation ratio parameter characterizing the fold change of transcript abundance over the length of the DNA region interrogated by the probes.

### 3′/5′-controls are affected by the hybridization mode

It was previously shown that the 3′/5′ intensity ratios of special control probe sets interrogating long transcripts such as GADPH and beta-actin might not represent a sufficient measure of the degradation bias at small expression degrees because non-specific binding leads to an underestimation of the 3′/5′-bias [34]. Here we show that the controls are often prone to saturation which also leads to the systematic underestimation of the 3′/5′-bias (see also Eq. (6)).

**Figure 7:**
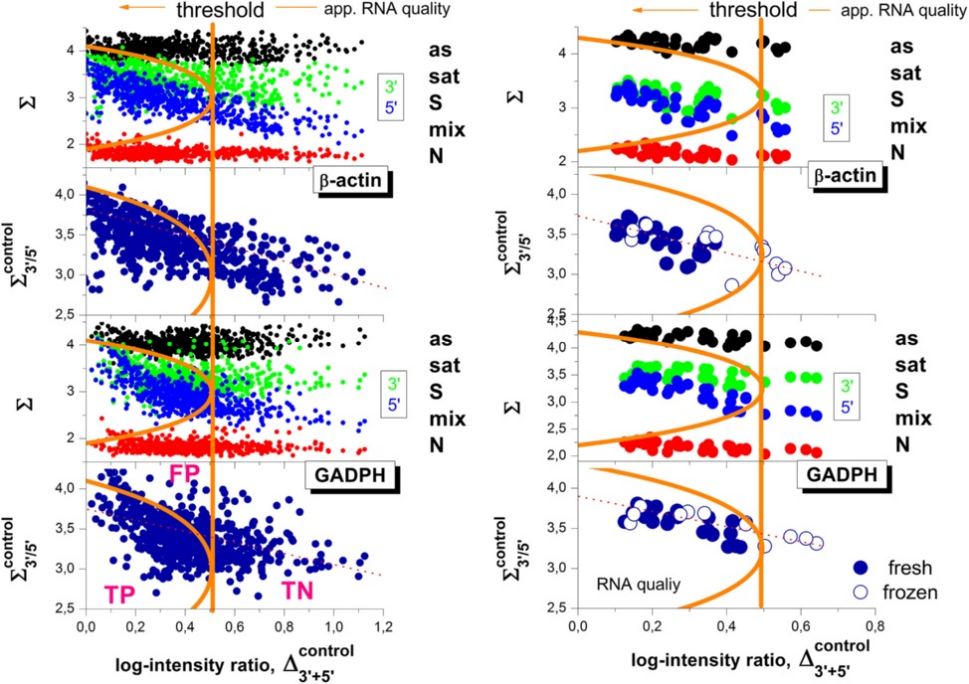
**Hybridization and RNA-quality characteristics of the GADPH and beta-actin control probe sets in the tissue (left) and rat-QC (right) data sets.** Each data point refers to one array of the respective series. The abscissa provides the degradation level in units of the logged 3′/5′- mean intensity ratio of the respective control data sets. The vertical axes plot either sigma coordinates of the 3′- (green dots) and 5′- (blue dots) probe sets of the controls, or their mean (dark blue circles). The red and black dots mark the respective sigma-levels of non-specific binding and of saturation, respectively. The vertical orange lines indicate the constant quality threshold separating good (to the left) and poor (to the right) apparent RNA quality. The ‘threshold’ hooks (orange) refer to the same quality threshold. They however explicitly consider its decrease in the N- and sat-ranges of hybridization. Application of the constant threshold thus produces false positives together with true positives and true negatives (see also Figure 12)

In the first step we estimated the hybridization regime of the GADPH and beta-actin controls of the rat-QC and tissue data sets using modified hook plots (Figure 7). They depict the logged PM-intensity ratio of the 3′- and 5′-probe sets of the controls (Δ_3′/5′_^control^, Eq. (25)) along the horizontal coordinate and either the sigma coordinate of each probe set (Σ, Eq. (14)) or the mean sigma of both probe sets (Σ_3′+5′_^control^, Eq. (25)) along the vertical coordinate axis. In the former plots, each control (GADPH and beta-actin) thus provides two data points per array referring to the 3′- and 5′-probe sets, respectively (see green and blue dots in Figure 7). In the latter plots both data points are merged together to illustrate the mean intensity trend of the controls as a function of the degradation index.

To judge the hybridization mode we also depict the sigma coordinates of the non-specific background intensity (N, red dots) and of the asymptotic saturation level (as, black dots) obtained from the standard hook analysis of each of the arrays. Recall that the sigma-values of the N- and the as-mode limit the range of possible probe intensities. They consequently constitute an intrinsic metric allowing to assign the probes to one of the five possible hybridization modes as indicated in the figure (see also Figure 3 and ref. [33,34]). It turned out, that small values of the degradation index (Δ_3′/5′_^control^ < 0.2) are often associated with sigma-values near the asymptotic saturation limit of the intensities especially in the tissue data set. We argued above that intensity-based degradation measures are not suited to exactly estimate the degradation level in the saturation limit of the probes. In best case they underestimate the true degradation level; in worst case the intensity ratios become meaningless.

It has been recommended that good-quality samples should have a 3′/5′ signal ratio for GADPH and beta-actin of no more than three, or in our notation of Δ_3′/5′_^control^ < log(3) = 0.47 [31]. We display this threshold as the vertical orange line in both parts of Figure 7. It consequently divides the data points of each data set into (apparently) bad and good ones for Δ_3′/5′_^control^ > threshold and Δ_3′/5′_^control^ < threshold, respectively.

The 3′/5′-intensity ratio of the probe sets is however not a constant for a given RNA-quality level. Instead it depends on the hybridization mode (see above and Eq. (6)). Particularly, the 3′/5′-intensity ratio referring to a constant RNA-quality level follows the degradation hook shown in Figure 3: It is maximal in the S-hybridization range and vanishes near the N- and as-ranges of hybridization. We plot representative degradation hook curves in Figure 7 (see the orange curves; note that the x- and y-axes are exchanged in comparison with Figure 3) which are calculated using the threshold value of good RNA-quality (Δγ_3′/5′_ = 0.47, see Eqs. (14) and (24)) the mean sigma-levels in the N- and as-ranges of the respective data sets. Hence, the degradation hook illustrates that the threshold value of the 3′/5′-intensity ratio strongly decreases in the mix- and sat-ranges due to the progressive effects of non-specific hybridization and of saturation, respectively. It consequently defines a variable, sigma-dependent threshold-curve which allows to differentiate between bad and good RNA quality data independent of the particular hybridization mode of the respective probes. In other words, it is more appropriate to apply this variable 3′/5′-‘threshold hook’ for quality assessment beyond the linear hybridization range instead of using a constant threshold value of the 3′/5′-intensity ratio.

For example, a large fraction of the GADPH- and beta-actin intensity ratios of the tissue data set meet the constant quality criterion, Δ_3′/5′_^control^ < threshold = 0.47, indicating apparently good RNA quality (Figure 7, right part). Consideration of the hybridization-dependent ‘threshold hook’ divides this region further into true positive estimates (Δ_3′/5′_^control^ < hook_threshold_) and false positives (hook_threshold_ < Δ_3′/5′_^control^ < threshold), where the latter data are located between the curved and linear thresholds as shown in Figure 7. We estimated a positive predictive value for GADPH controls of about 0.48 which reflects overestimation of RNA-quality for about 50% of all 677 arrays of the tissue data set (see Methods section). Note also that strong saturation of the probes can completely prevent detection of poor RNA-quality samples because the respective intensity ratio levels off to Δ_3′/5′_^control^ = 0.

The mean sigma coordinates (Σ_3′+5′_^control^) of the Rat-QC data set are found approximately halfway between the respective N- and as- levels indicating that the controls are predominantly hybridized in the S-range (Figure 7, left part). Application of a constant quality threshold seems appropriate for this data.

The sigma (i.e. logged mean intensity values) values of both data sets studied clearly indicate the decrease of the mean intensity of the controls with decreasing RNA quality due to the loss of material assumed, e.g. in Eq. (13). In consequence, the hybridization regime of the controls can shift with changing RNA-quality. Note also that GADPH is associated with slightly larger probe signals than beta-actin in both data sets. Beta-actin controls are consequently less prone to saturation than GADPH controls.

In summary, control probes can overestimate RNA-quality if one uses a constant threshold criterion because the true threshold level strongly decays for saturated probes. The problem can be fixed either by using an intensity dependent ‘threshold’ hook or by using alternative RNA-quality estimates such as the degradation ratio (see above).

### Affy-slope is affected by absent probes

A widely applied metric for RNA quality is the ‘RNA degradation plot’ provided with the R package *affy*[15,16]. The RNA degradation plot displays the mean log intensity averaged over all probes with the same index k of one microarray as a function of the probe index, k = 1,…,N_pset_. The slope of the regression line then provides a summary measure to characterize the mean degree of RNA-degradation in a chip-specific fashion. Note that the affy-slope parameter originally does not intend to serve as an absolute RNA quality measure per se but instead, represents a relative measure for comparing RNA quality between different chips in a particular series of measurements.

However, the affy-slope degradation plot is virtually identical with the reciprocal positional dependent degradation index introduced above in Eq. (9) (d^h^(k)^-1^) except the fact that it considers all probes of the array whereas our approach separately averages over the N- and S-subensembles referring either to the S- or N-hybridization regimes, respectively. The affy-slope estimates are expected to underestimate the degradation level owing to the inclusion of predominantly non-specifically hybridized probes (so-called absent probes) which do not respond to RNA quality as shown above. More importantly, the chip-to-chip variability of the fraction of absent probes (%N; as determined by methods such as MAS5 or hook; see ref. [34] for comparison) is expected to affect the affy-slope measures by factors which are not or only weakly related to RNA quality.

**Figure 8:**
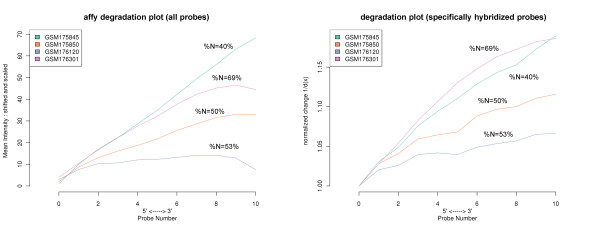
**RNA degradation plot of all probes (panel a) and degradation profile of specifically hybridized probes (b) for microarrays selected from the human tissue data set.** Panel **a** shows the plots obtained using the affy package [16] whereas the curves in panel **b** are given by the inverse of the degradation function d^S^(k)^-1^ (see Eq. (10)). The slopes of most of the curves rank in the same order in both panels, except the two curves of steepest slope which reverse order in both parts of the figure owing to the different percentage of absent probes. The percentage of absent probes are %N = 40% (GSM175845), 69% (GSM176301), 50% (GSM175850) and 53% (GSM176120) as determined by the hook method [33,34]

To illustrate this effect, a series of affy-slope curves referring to different degradation levels are shown in Figure 8a. Panel b of the figure plots our degradation profiles d^S^(k) of the specifically hybridized probes for the same arrays. Both presentations provide similar trends for the microarrays with similar %N-values. However, affy-slope and our degradation plot provide different results for arrays with marked differences of %N, as expected. Particularly, affy-slope tends to underestimate the slope for large %N values and thus to overestimate RNA quality.

To generalize this result we studied the mean 5′/3′-intensity ratios of different subsets of probes taken from the 677 array hybridizations of the human tissue data set ( Additional file 1). We found that the affyslope-measure systematically increases with the amount of specific hybridization and with decreasing amount of absent probes per array. The more detailed evaluation of the functional relation between the ‘true’ degradation index estimated in the S-hybridization regime and the apparent one obtained from all probe sets predicts a linear dependence between the apparent and the true degradation ratios where the slope is expected to decrease with increasing %N ( Additional file 1). Theory also predicts that the apparent 5′/3′-ratio directly varies with the fraction of specifically hybridized probe sets in agreement with the tissue data set.

Hence, the apparent degradation ratio derived from the simple affy-slope intensity measures is strongly modulated by the fraction of non-specifically hybridized ‘absent’ probes leading potentially to the systematic overestimation of RNA quality. Contrarily, the proposed use of specifically hybridized probes largely removes this bias from the data and provides a reliable measure of the degradation degree which can be consistently compared between different arrays.

### Array-degradation metrics correlate with RIN

**Figure 9:**
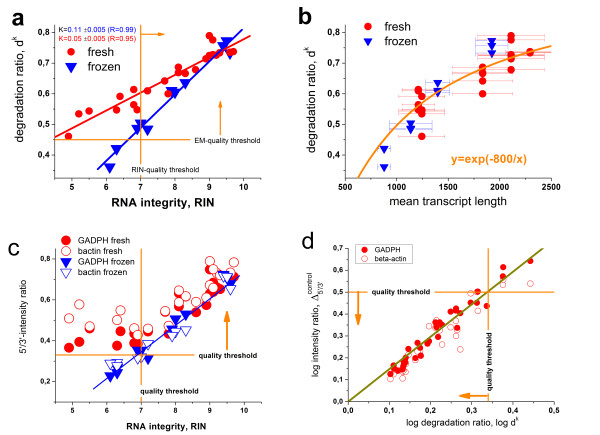
**Comparison of microarray degradation measures (d**^**k **^**and 5′/3′-ratio of the hybridization controls) with the RNA integrity number (RIN, panel a and c) and with the mean length of the transcripts (panel b) obtained in the ratQC experiment. **[10] The d^k^ parameter split into two branches for the two sample treatments when plotted as a function of RIN whereas the d^k^ data virtually merge into one branch if plotted as a function of transcript length. Panel **d** correlates the d^k^ and the 5′/3′- intensity ratio of the control probes in logarithmic scale. The vertical and horizontal orange lines indicate the respective quality thresholds. Good RNA-quality probes are found in direction of the arrows

The RNA Integrity Number (RIN) provides a numerical value for the assessment of RNA quality based on the electropherogram trace of a RNA sample captured with the Agilent Bioanalyzer [13]. The RIN is widely used and its scale ranges from 1 to 10 (most to least degraded). A RIN-cutoff of RIN ≥ 7 is recommended for obtaining good-quality RNA for microarray analysis [32]. Figure 9a compares our d^k^ degradation measure with the RIN reference values obtained in the ratQC experiment. Both measures correlate strongly, however, the two samples and incubation conditions result in different slopes of the regression lines. In other words, each microarray degradation parameter does not unambiguously transform into one RIN-value especially at larger degradation levels. Instead, the two different samples and incubation conditions reflect a bimodal relation between the two types of measures: each RIN value splits into two d^k^ options and vice versa. Note also that correlation coefficient between RIN and our improved d^k^-degradation measure exceeds that between RIN and affyslope (r = 0.95 vs 0.92, RatQC fresh) owing to the reasons discussed in the previous subsection.

In panel b of Figure 9 we re-plot the d^k^ degradation parameter as a function of the mean transcript length measured independently using the Bioanalyzer [10]. The two branches of the d^k^ -vs-RIN plot merge into one within the error limits. This result confirms that our microarray-based degradation measure more directly relates to the mean transcript length and thus to the state of the mRNAs that the microarray experiment intends to quantify. The RIN however represents an alternative integrity measure capturing a series of electropherogram features that are indicative also for additional properties of the RNA solution such as the ratio of larger to smaller molecules and how far the degradation process has proceeded [10]. The correlation of the 5′/3′ ratios of the degradation controls with the RIN-numbers reveals subtle differences compared with the behavior of the d^k^ parameter (Figure 9c). Particularly, the hybridization control-measures taken from the ‘fresh’ samples are virtually independent of degradation at RIN < 7 whereas for RIN > 7 both treatments give rise to similar behavior of the controls. Recall that the GADPH and beta-actin controls cover a slightly wider range of the transcripts (from about 200 to about 1000 nt, see Figure 2b) than the d^k^ degradation ratio which probes the range from about 100 to about 550, on the average (see Figure 2c). This difference presumably explains the smaller values of the control ratios at larger expression degrees. More importantly, the d^k^ parameter is calculated as the average over a large number of probes. Presumably both types of parameters respond differently to changes of the length distribution of the transcripts due to degradation. Below we address this issue more in detail.

The regression line between the d^k^ parameter and the 5′/3′-intensity ratio of the controls allows to transform the quality threshold of the latter ratio into a d^k^ threshold (Figure 9d, see orange lines). We replot these thresholds into panel a and c of Figure 9: The RIN threshold is clearly more restrictive assigning more samples to bad RNA-quality than the threshold of the microarray-based control probes.

### Degradation reduces total transcript abundance

We so far estimated RNA quality in relative units using suited 5′/3′-intensity metrics which reflect the decrease of transcript abundance with increasing distance from the 3′-end. Trivially, this effect is expected to reduce the total amount of mRNA used for hybridization. The decrease of the mean intensity of the control probe sets with increasing degradation ratio as shown in Figure 7 confirms the decrease of the total amount of the respective specific transcripts with progressive degradation. Figure 7 also shows the non-specific intensity level of each of the arrays studied (red dots), which tends to decrease with increasing degradation.

**Figure 10:**
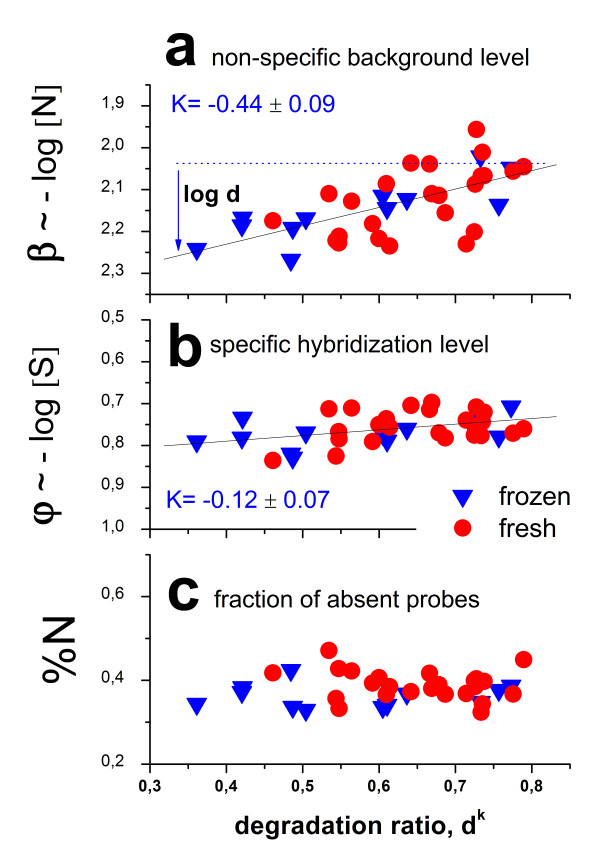
**Hook-hybridization characteristics of the arrays of the ratQC data set.** (**a**) The width of the hook curves β increases with progressive degradation indicating the decrease of the non-specific background due to the loss of material (see Eq. (18)). Log d is the mean degradation index (Eq. (2)) and K the slope of the regression line. The mean level of specific hybridization changes only weakly with degradation (**b**). The fraction of absent probes is virtually unaffected by degradation (**c**). All parameters are estimated using the hook method [33,34]

The hook method enables the independent estimation of the mean levels of non-specific ‘background’ hybridization and that of specific expression using the simple summary measures β (see Eq. (18)) and ϕ (Eq. (19)) which are based on large ensembles of probe sets on each array. Particularly, the width of the hook curve β has been shown to relate to the total amount of RNA material [30,34]. The width of the hook curves clearly decreases with progressive degradation. The observed decrement indicates that the amount of RNA material decreases by about 40% in the Rat-QC experiment (see Figure 10a).

The degree of specific binding drops upon degradation, however to a considerable smaller degree than the amount of non-specific binding (Figure 10b). This discrepancy surprises because naively one expects that the loss of material similarly affects specific and non-specific binding on the average, i.e. d^N^ ≈ d^S^. The mean hybridization levels of specific and non-specific binding are however directly related also to the respective mean binding constants, <K^P,S^ > and < K^N^>, respectively (Eq (4)). We have previously shown, that the decrease of RNA-material used for hybridization increases the specific binding constant due to weaker bulk hybridization and vice versa [30]. In consequence, this so-called up-down effect will partly compensate the decrease of the concentration of specific transcripts giving rise to the smaller decrease of the specific hybridization strength upon RNA degradation.

Part c of Figure 10 depicts the percentage of absent probes detected on each of the arrays. It remains essentially unaffected by RNA degradation. This result shows that the loss of material does not affect the detection threshold of the array experiment for specific binding.

### RNA-quality scaling of gene expression

It has been previously found that, although moderate levels of RNA degradation are tolerated by differential expression analysis, beyond a threshold especially long targets provide erroneous expression results [10,38]. Systematic large-scale microarray analyses reveal that the expression values of up to 30% of all genes probed on an array significantly correlate with degradation quality measures such as the 3′/5′-ratios of control genes [4,21]. The observed correlations can be well explained on the basis of the results presented here: For example, it is found that the expression values of weakly expressed genes negatively correlate with the quality of their transcripts [4]. The authors explain this ‘…the worse the quality the stronger the signal…’-effect by either the enrichment of low quality RNA in the low signal range due to nonspecific hybridization or by compensating effects due to chip-to-chip normalization. The former interpretation disagrees with our results presented in the previous subsection. We found that progressive degradation dilutes the sample and this way decreases the amount of nonspecific hybridization. On the other hand, the observed negative correlations also mean ‘…the better the (apparent) quality the weaker the signal…’ in agreement with our results: For low intensity signals the 3′/5′-ratio indeed improves with decreasing intensity suggesting better RNA-quality. We demonstrated that this trend is however caused by the increasing amount of nonspecific hybridization and not by improved RNA-integrity.

Considering also correlations between 3′/5′-quality measures and signal values (called LEV, ‘labeling extension values’), Lee et al. [21] found that LEV are typically small at low expression values but step-wisely increase beyond a certain expression threshold. The authors hypothesized that the positional 3′/5′-bias is less notable for low abundant transcripts due to inefficient reverse transcription. However, according to our results, the observed trend can be explained by the dominance of non-specific hybridization lacking positional 3′-bias at small expression levels. These two examples demonstrate advantages of model-based expression analysis using physico-chemical hybridization theory compared with simple correlation analysis.

The final aim is therefore to use the degradation model for correcting the 3′-probe intensity bias to provide (largely) unbiased probe signals for downstream analysis. One expects that the loss of RNA material in general and particularly, RNA-fragments probed far away from the 3′-end, systematically decreases the apparent expression degree extracted from microarray probe intensities. In the Methods-section we propose a simple algorithm which corrects the raw probe intensities for the positional-dependent 3′/5′-bias. It makes use of the mean intensity decay-function of specifically hybridized probes and of the degree of specific hybridization of the particular probe which it intends to correct. Our approach corrects the 3′/5′-bias on the level of raw probe intensities.

 In the supplementary text ( Additional file 1) we compare two correction metrics based either on the absolute probe position (‘L-correction’) or on the relative probe position (‘k-correction’) relative to the 3′-transcript end. The k-correction applies the same factor to all probe sets. Their mean intensity is effectively scaled solely by the degree of specific hybridization (see the Methods section). Contrarily, the L-correction applies a specific factor to each probe-set depending on the mean absolute position of the respective probes. The comparison of both correction methods shows that probe sets located on the average nearer to 3′-end of the transcript are corrected to a less degree using their absolute position than probe sets located more distant from the 3′-transcript end. Hence, the L-correction is more specific with respect to each particular probe set. On the other hand, the k-correction is more robust with respect to outliers.

## Conclusions

Amplification of RNA-material using primed in-vitro transcription protocols and degradation of RNA during extraction, storage and processing of the samples affects RNA-quality in microarray experiments with consequences for expression estimates and their interpretation. We systematically analysed the effect of varying RNA quality on microarray probe intensities using a physico-chemical hybridization model and propose (i) new measures to assess RNA quality and (ii), a simple method to correct probe intensities for the degradation bias.

Particularly, it is shown that

– poor RNA quality is associated with a 3′-bias of transcript abundance which affects only the probe signal due to specific hybridization;

– estimation and correction of the signal bias of each particular probe requires consideration of its hybridization mode (specific, non-specific or a superposition of both) and of the positional effect of probe intensity along the respective gene due to truncated transcripts. The former issue is solved by applying a modified ‘hook’-approach of data analysis based on Langmuir hybridization theory. The latter effect is taken into account by estimating the mean positional intensity decay on each array as a function of either the probe index or the probe’s distance to the 3′ end of its target transcript.

– RNA quality is estimated in terms of the 3′/5′-intensity gradient of specifically hybridized probes. In addition to appropriate quality numbers (such as ‘tongs opening’-parameter and the degradation ratio) we introduce graphical characteristics allowing assessment of RNA quality of each single array (‘tongs plot’ and ‘degradation hook’). The parameters have a well defined physical meaning related to the fold change of transcript abundance along the genes. ‘Poor’ RNA quality is characterized roughly by a decay of the mean specific signal by a factor of less than 0.5 between probes near the 3′- end and probes located about 600 nt away.

– our approach improves established RNA-integrity measures such as ‘affyslope’ and the 3′/5′-intensity ratio of degradation control probe sets. Both methods are prone to overestimate RNA quality if the signals are dominated by non-specific hybridization (affyslope) and/or saturation (controls). Our microarray-based quality estimate correlates well with the RNA integrity number (RIN) which, in addition, is affected by more complex properties of RNA degradation not uniquely related to transcript length.

– short probe sets near the 3′-end are prone to non-specific hybridization presumable because of uncertainties in 3′UTR length owing to inaccurate assignment of the 3′-end and transcript isoforms.

– poor RNA quality is associated with a decreased amount of RNA material hybridized on the array paralleled by a decreased total signal level. Additionally, it causes a gene-specific loss of signal due to the positional bias of transcript abundance which requires an individual, gene-specific correction. The former total effect can decrease the overall signal level of an array by the factor of 0.5 -0.7 in the case of poor RNA quality (RIN < 7). The latter local effect can be more pronounced with a penalty in expression measures by a factor of 0.3-0.4 or even less in worst cases.

Our study proposes new degradation measures to judge RNA quality. The basic difference to previous ones is the explicit consideration of the hybridization mode to largely reduce the influence of non-specific hybridization. Based on the physico-chemical mechanisms of probe hybridization we propose a correction method which aims at removing the degradation bias and thus enables to use the full ensemble of probes in downstream analysis without loss of information.

The tongs visualizations and the degradation measures are implemented in the Bioconductor package *AffyRNADegradation*. Probe distance files are available under the website http://www.izbi.uni-leipzig.de/downloads_links/programs/rna_integrity.php.

## Methods

### Data

Affymetrix microarray raw intensity data (CEL-file format) were downloaded from the public repositories Gene expression omnibus ( http://www.ncbi.nlm.nih.gov/geo) or Array Express ( http://www.ebi.ac.uk/microarray-as/ae/). We studied the following data sets:

The *Human tissue* dataset (GSE7307, see supplementary text for the detailed list of samples used) comprises 677 samples taken from over 90 distinct tissue types hybridized to Affymetrix HG-U133 plus 2.0 arrays.

The *RatQC* (rat quality control) dataset (E-MEXP-1069) from ref. [10] was generated to systemically explore how RNA quality affects microarray results. It consists of 36 rat liver RNA samples hybridized to Affymetrix RAE230A expression arrays. The progressive change in RNA quality was generated either by thawing frozen tissue or by ex vivo incubation of fresh tissue. Each sample was characterized by the RNA integrity number (RIN) and mean transcript length in ref. [10].

The *RNeasy* data set consists of five pairs of HG-U133A GeneChips which were hybridized with RNA extracted from ovarian cancer samples and processed in two different ways namely with and without a cleanup step using RNeasy reagents [3]. The RNeasy cleanup should lead to good-quality RNA whereas lack of the cleanup step should yield poorer-quality RNA. The RNeasy data set was used in previous work aiming at judging RNA-quality from microarray data [39,40].

Probe set definitions were applied as proposed by Affymetrix by using standard CDF-files. Absolute probe positions with respect to the intended 3′-end of the transcript, L_p_, (as used, e.g. in Eq. (8)) were determined from the target sequences provided by Affymetrix for each transcript (see http://www.affymetrix.com) by aligning the probe sequences to the respective transcript sequence. The position of each probe L_p_ (p = 1, 2…) is then defined as the number of nucleotides counted between the 3′-end of the transcript and the first (i.e. nearest) base of the 25meric probe sequence.

### Degradation hook and tongs plot

The so-called hook method aims at characterizing the hybridization of a particular microarray in terms of quality control and expression analysis (see [33,34,41] for a detailed description). This single-chip method applies to microarrays of the GeneChip-type containing pairs of perfect match (PM) and mismatch (MM) probes. The method processes PM and MM probe intensities after sequence correction (I^PM,corr^ and I^MM,corr^, respectively) using the transformations

(14) $$\begin{array}{l} \Delta \equiv {〈\Delta_{p}〉}_{\text{mov}}\;\text{and}\;\Sigma \equiv {〈\Sigma {}_{p}〉}_{\text{pset}}\\ \text{with}\;\Delta_{p}=\log\;I_{p}^{\text{PM,corr}}-\log\;I_{p}^{\text{MM,corr}}\\ \text{and}\;\Sigma {}_{p}=\frac{1}{2}\left(\log\;I_{p}^{\text{PM,corr}}+\log\;I_{p}^{\text{MM,corr}}\right) \end{array}$$

<… > _pset_ denotes averaging over each probe set of usually 11 PM/MM probe pairs addressing the same transcript (log ≡ log_10_ is the decadic logarithm) and < … > _mov_ is the moving average over a sliding window of about 1000 probes for smoothing. Plotting the data into Δ-versus-Σ coordinates provides the hook curve which enables decomposition of the probe signals into contributions due to specific and non-specific hybridization by simple visual inspection.

In analogy with Eq. (14) we define the modified ordinate values

(15) $$\begin{array}{l} \;\Delta \Sigma {}_{S}\equiv {〈\Sigma {}_{p}〉}_{S}-\Sigma \;\text{and}\\ \Delta \Sigma {}_{3^{\prime }/5^{\prime }}\equiv {〈\Sigma {}_{p}〉}_{3^{\prime }}-{〈\Sigma {}_{p}〉}_{5^{\prime }} \end{array}$$

where < … > _s_ denotes averaging over subsets of three probes from the respective probe set located either nearer its 3′-end (s = 3′), 5′-end (s = 5′) or in the middle (s = m), and subsequent smoothing using a sliding window of appropriate size. The so-called tongs-plot shows the three positional-dependent values ΔΣ_3′_, ΔΣ_5′_ and optionally ΔΣ_m_ as a function of the hook-abscissa Σ whereas the ‘degradation hook’ plots ΔΣ_3′/5′_-versus- Σ.

The two-species Langmuir hybridization isotherm predicts the theoretical hook-curve which was previously used to fit the experimental curves and to extract characteristic chip-related parameters. It provides a ‘mean’ hybridization isotherm implicitly characterizing the concentration dependence of the probe signals. Here we modify the hook formalism to take into account the effect of incomplete transcript amplification and degradation in terms of the degradation ratios defined in Eq. (2). For the detailed derivation of the hook equation in the absence of degradation and the detailed discussion of the used parameters see refs. [33] and [34]. The theoretical expressions for the ‘degradation’ hook and the tongs-plot are obtained analogously.

In short, insertion of Eqs. (3) and (4) with P = PM and MM and w_p_^P,h^ = 1 into Eqs. (14) and (15) provides the theoretical expressions of the hook coordinates for the subset s of probes taken from the probe sets,

(16) $$\begin{array}{l} {〈\Delta_{p}〉}_{s}\equiv \Delta \left(R_{S}\right)=\log\left\{\left(R_{s}+1\right)/\left(R_{s}{\times 10}^{-\alpha }+1\right)\right\}\\ \;-\log\left\{B^{\text{PM}}\left(R_{s}\right){\text{/B}}^{\text{MM}}\left(R_{s}\right)\right\}\text{and}\\ {〈\Sigma {}_{p}〉}_{s}\equiv \Sigma \left(R_{s}\right)=\Sigma {}^{\text{start}}+\frac{1}{2}\log\left\{\left(R_{s}+1\right)\right)\\ \;\left(\times ,\left(R_{s}{\times 10}^{-\alpha }+1\right)\right\}-\frac{1}{2}\log\left\{B^{\text{PM}}\left(R_{s}\right){\text{×B}}^{\text{MM}}\left(R_{s}\right)\right\} \end{array}$$

with the saturation terms

(17) $$\begin{array}{l} B^{\text{PM}}\left(R_{s}\right){\text{=1+}10}^{-\left(\text{β+}\frac{1}{2}\text{Δstart}\right)}\cdot \left(R_{s}+1\right)\;\text{and}\\ B^{\text{MM}}\left(R_{s}\right){\text{=1+}10}^{-\left(\text{β+}\frac{1}{2}\text{Δstart}\right)}\cdot \left(R_{s}\cdot 10^{-\text{α}}+1\right) \end{array}$$

Eq. (16) expresses the hook-coordinates as a function of the probe-specific S/N-ratio,

(18) $$\begin{array}{l} R_{s}=\frac{{〈d_{p}〉}_{s}}{d}\cdot \frac{X^{\text{PM,S}}}{X^{\text{PM,N}}}=\frac{{〈d_{p}〉}_{s}}{d}\cdot \frac{{\left[s\right]}_{\text{target}}}{{\left[\text{N}\right]}_{\text{chip}}}\\ \;\cdot \frac{{〈{\text{Κ}}_{p}^{\text{PM,S}}〉}_{\text{chip}}}{{〈{\text{Κ}}_{p}^{\text{PM,N}}〉}_{\text{chip}}} \end{array}$$

It scales with ${〈d_{p}〉}_{s}$/d, the probe specific 3′-bias of the actual transcript abundance averaged over the subset s and divided by the mean degradation index of the selected chip, d. The standard version of the hook (Eq. (14)) is described by Eq. (16) with ${〈d_{p}〉}_{s}/\text{d=}\;1$ because averages are calculated over all probes of each probeset (s = pset).

The vertical and horizontal dimensions of the hook curve and its start coordinates are defined as

(19) $$\begin{array}{l} \alpha \approx \log\frac{{〈K_{\rho }^{\text{PM,S}}〉}_{\text{chip}}}{{〈K_{\rho }^{\text{PM,S}}〉}_{\text{chip}}},\;\beta \approx -\left(\text{logd+}{〈{\text{logX}}_{\rho }^{\text{PM,N}}〉}_{\text{chip}}\right)\\ \text{and}\;\Sigma {}^{\text{start}}\;=\;\text{logM}\;+\;{〈{\text{logX}}_{\rho }^{\text{PM,N}}〉}_{\text{chip}}\text{+logd} \end{array}$$

respectively. Note that the width and the start coordinate of the hook curve, β and Σ_start_, change with the mean degradation index d (see also Eq. (2)) whereas the height of the hook α doesn’t depend on degradation.

The mean expression index characterizes the mean expression level of present probes of the chip,

(20) $$\begin{array}{l} \phi \equiv {〈\text{log}\left(d_{p}^{\text{M,S}}{\text{×X}}_{p}^{\text{M,S}}\right)〉}_{\text{chip}}\\ \sim {〈\text{log}\;\left(R\right)+\log\;X_{p}^{\text{M,N}}+\log\;d_{p}^{\text{M,S}}〉}_{\text{chip}} \end{array}$$

The ordinate values of the degradation plots are obtained by inserting Eq. (16) into Eq. (15),

$$\begin{array}{l} \Delta \Sigma {}_{s}\left(R\right)\equiv \Sigma \left(R_{S}^{S}\right)-\Sigma \left(R\right)\;\text{and}\\ \Delta \Sigma {}_{s1/s2}\left(R\right)\equiv \Sigma \left(R_{s1}\right)-\Sigma \left(R_{s2}\right) \end{array}$$

One gets after explicit consideration of Eq. (16)

(21) $$\begin{array}{l} \text{Δ}\Sigma {}_{\text{s1/s}2}\left(R\right)=\frac{1}{2}\text{log}\frac{\left(R_{s1}+1\right)\text{×}\left(R_{s1}{\text{×}10}^{-\text{α}}+1\right)}{\left(R_{s2}+1\right)\text{×}\left(R_{s2}{\text{×}10}^{-\text{α}}+1\right)}\\ \;-\frac{1}{2}\text{log}\frac{{\text{B}}^{\text{PM}}\left(R_{s1}\right){\text{×B}}^{\text{MM}}\left(R_{s1}\right)}{{\text{B}}^{\text{PM}}\left(R_{s2}\right){\text{×B}}_{p}^{\text{M,M}}\left(R_{s2}\right)} \end{array}$$

where ΔΣ_s1_(R) refers to the special case r_s2_ = 1. The parameters

(22) $$\begin{array}{l} {\text{γ}}_{s}\equiv {\text{logr}}_{s}\;=\;\text{log}\frac{{〈\left[s\right]〉}_{s}}{{〈\left[s\right]〉}_{\text{chip}}}\;\text{with}\;\text{s=}3^{\prime },5^{\prime }\text{,m}\;\text{and}\\ {\text{Δγ}}_{3^{\prime }\text{/}5^{\prime }}\equiv {\text{γ}}_{3^{\prime }}{-\text{γ}}_{5^{\prime }}\;=\;\text{log}\frac{{〈\left[s\right]〉}_{3^{\prime }}}{{〈\left[s\right]〉}_{5^{\prime }}} \end{array}$$

define the 3′-bias of transcript abundance (see also Eqs. (17) and (6)). Particularly, $\Delta_{\text{γ}}{}_{3\text{'/5'}}$ provides the logged fold change of the probe specific transcript concentrations between probes located nearer the 3′- and 5′-ends of the transcript. The mean transcript concentration averaged over all probes can be estimated as the geometric mean over the 3′ and 5′ transcript concentrations,

(23) $$\begin{array}{l} {〈s〉}_{\text{chip}}\approx {〈\left[s\right]〉}_{3^{\prime }}10^{-\frac{1}{2}\Delta \gamma 3^{\prime }/5^{\prime }}\approx {\left[s\right]}_{3^{\prime }}\cdot \sqrt{\frac{{\left[s\right]}_{5^{\prime }}}{{\left[s\right]}_{3^{\prime }}}}\\ \;\approx \sqrt{{\left[s\right]}_{3^{\prime }}\cdot {\left[s\right]}_{5^{\prime }}} \end{array}$$

if one assumes uniformly distributed probes along the relevant transcript regions. With ${\left[s\right]}_{\text{target}}\approx {〈\left[s\right]〉}_{3'}$ and Eq. (2) one gets

(24) $$\log d\approx -0.5\cdot \Delta {\text{γ}}_{3^{\prime }/5^{\prime }}$$

Hence, the mean amount of RNA (Eq. (2)) is directly related to the 3′/5′-difference of transcript abundance.

**Figure 11:**
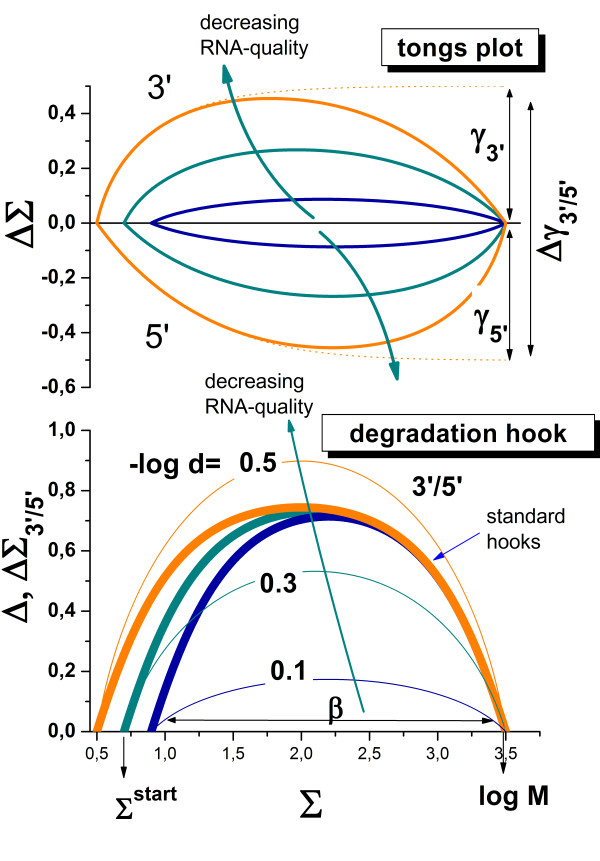
**Theoretical hook curve (Eq. (16), thick curves), degradation hook (thin curves) and tongs plot (panel above; Eqs. (20) and (21)) for different degradation levels log d.** With increasing degradation the positive and negative amplitudes of the tongs plot (the tongs opening Δγ_3′/5′_) and the height of the degradation hook increase, accompanied by the shift of its increasing branch towards the left which widens the curves (parameter β). The curves are calculated with γ_3′_ = −γ_5′_ = 0.1, 0.3 and 0.5, respectively. The dotted curves in the part above are calculated neglecting the saturation term in Eq. (21). The geometrical meaning of selected parameters is indicated by arrows (see text)

Typical examples of the hook curve (Δ-versus-Σ, Eq. (16), thick curves), the degradation hook (ΔΣ_3′/5′_-versus-Σ, Eq. (21), thin curves) and the tongs-plot ((ΔΣ-versus-Σ, Eq. (21), panel above) as predicted by theory are shown in Figure 11 for different degradation levels. Increasing degradation increases the opening of the tongs and widens the hook. Both changes are governed by the degradation ratio d and r_s_ and their logarithmic transformations (see Eqs. (2), (18), (22) and (24)). The widening of the hook by –log d reflects the decrease of the mean transcript concentration due to incomplete amplification and degradation. This trend is equivalent with the decrease of the mean level of non-specific background hybridization which in turn increases the mean binding constant of specific binding [30]. The consequences of this so-called up-down effect are discussed above.

### Threshold hook

The threshold hook represents a special version of the degradation hook described in the previous subsection. It defines a threshold of the 3′/5-intensity ratio of the probe sets used to assess RNA-quality such a GADPH or beta-actin. The threshold hook accounts for the fact that the probe signals are affected by non-specific binding and by saturation. Both effects give rise to an intensity-dependent threshold for estimating good RNA-quality.

In the first step one transforms the intensity values of the control probes into hook coordinates

(25) $$\begin{array}{l} \sum {}_{3^{\prime }+5^{\prime }}^{\mathrm{control}}=\left({〈\log I^{\mathrm{PM}}〉}_{3^{\prime }\text{probeset}}+{〈\log I^{\mathrm{PM}}〉}_{5^{\prime }\text{probeset}}\right)\\ \log d_{3^{\prime }/5^{\prime }}^{\mathrm{control}}\equiv \Delta_{3^{\prime }/5^{\prime }}^{\mathrm{control}}={〈\log I^{\mathrm{PM}}〉}_{3^{\prime }\text{probeset}}-{〈\log I^{\mathrm{PM}}〉}_{5^{\prime }\text{probeset}} \end{array}$$

In contrast to the standard hook (Eq. (14)) we here use only the intensities of PM-probes.

In the second step, one calculates the threshold hook as a Δ_3′/5′_-vs-Σ_3′/5′_-plot under the condition that both, the 5′- and 3′-probes hybridize according to the hyperbolic Langmuir isotherm (see Eq. (3) with w_p_^P^ = 1), however using different specific binding activities due to the different degradation indices of the respective transcripts, d_5′_ < d_3′_ (Eq. (4)). The delta-value is expressed as a function of sigma using the degradation hook-formalism described in the previous subsection after neglecting the MM-probes (use Eq. (16) with α = −∞). The start and end point of the threshold hook are taken from the standard hook analysis which provides these data with relatively high accuracy.

**Figure 12:**
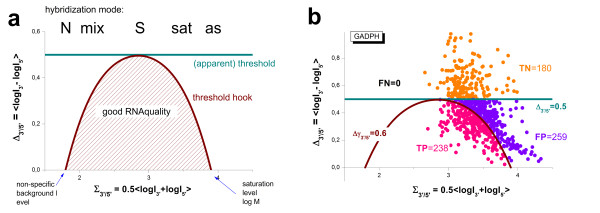
**Threshold hook for estimating good RNA quality using control probe sets.** (**a**) Constant (apparent) and variable (threshold hook) RNA quality threshold. The true threshold depends on the hybridization regime and vanished upon non-specific hybridization and upon saturation. (**b**) Error estimates of GADPH-controls taken from the tissue data set (see text)

The obtained hook curve thus describes the ‘trajectory’ of a pairing of 3′/5-probe sets upon changing expression degree of the respective transcript (see Figure 12a for illustration). Note that the delta-coordinate directly provides the apparent logged degradation ratio (Δ_3′/5′_ = −log(r_5′/3′_^app^), see Eq. (5)) whereas the ‘true’ degradation index is given by the height parameter used in the fits (Δγ_3′/5′_ = log(r_5′/3′_^true^), see Eq. (22)). The latter true degradation index is adjusted in such a way that the maximum value of the apparent degradation ratio agrees with the empirical RNA-quality threshold of the chosen control probe. Hence, the threshold hook transforms the constant RNA-quality threshold into a variable one which depends on the hybridization mode of the controls. In consequence, different data points residing along one hook curve refer to identical true degradation levels irrespective of their different delta coordinates characterizing their apparent degradation level.

The application of a constant threshold instead of the variable one will cause false quality estimates. We estimated the error of the 3′/5′-intensity ratio of the GADPH-control taken from the tissue data set as example: Figure 12b shows the hook-coordinates of the GADPH control probe sets of the 677 samples of the tissue data set (dots, see Eq. (25)). The threshold hook and the horizontal line provide the true and the apparent (false) thresholds for good RNA quality in terms of the logged 3′/5′-intensity ratios, Δ_3′/5′_ < Δ_threshold_. The constant threshold is assumed to agree with that of the hook curve in the range of specific hybridization. It consequently forms the tangent of the hook-curve at its maximum referring to the S-range of hybridization. The hook curve describes Δ_threshold_ under the realistic assumption of saturation whereas the constant threshold neglects this effect. As a consequence, data located between both thresholds (coloured in blue) define false positives (FP) with respect to the constant threshold whereas data below the hook and above the line are true positives (TP, red) and true negatives (TN, orange), respectively. The number of false negatives (FN) is zero because the hook threshold remains below the constant one. The positive predictive value (PPV = TP/(TP + FP)) and the specificity (SP = TN/(FP + TN)) are 0.48 and 0.79, respectively, meaning that less than 50% of the 3′/5′ controls properly estimate the quality of RNA in terms of good and degraded one. This particular example assumes that the 3′/5′ signal ratio for GADPH for good RNA is of no more than 3, or in our notation Δ_3′/5′_ < log(3) ≈ 0.5.

### Correcting the 3′/5′ bias of pobe intensities

RNA quality affects the probe intensities of each probe by two factors: (i) the probe position with respect to the nominal 3′-end of the transcript, L (or, alternatively, the probe index in the probe set, k) and (ii), the hybridization regime. The specific hybridization regime below saturation is particularly prone to biased intensities as opposed to non-specific hybridization and specific hybridization in the asymptotic saturation range. Our approach aims at correcting raw probe intensities which subsequently can be preprocessed using any method available.

Firstly, we estimate the degree of specific, unsaturated hybridization in terms of the relative amplitude of the degradation hook with respect to its maximum reached in the S-range of hybridization, ${\text{f}}^{s}\left(\text{y}\right)\text{=Δ}\sum {}_{3^{\prime }\text{/}5^{\prime }}\left(\text{y}\right)\text{/Δ}\sum {}_{3^{\prime }\text{/}5^{\prime }}^{\text{max}}$ using the logged PM-probe intensity averaged over each probe set as argument, i.e. $\text{y}\equiv 〈{\text{logI}}^{\text{PM}}〉$. Secondly, we estimate the decay function of the probe intensity for specifically hybridized probes, d^S^(x) (x = k, L), defined in Eqs. (9) and (10), for the respective array.

Then, the correction function

(26) $$\text{C}\left(\text{x,y}\right){\text{=d}}^{s}\left(X\right){\text{×f}}^{s}\left(\text{y}\right){\text{+d}}^{\text{N}}\left(X\right)\text{×}\left({1-\text{f}}^{s}\left(\text{y}\right)\right)$$

is defined as weighted sum of the decay functions due to specific and non-specific hybridization where the latter is set to unity, d^N^(x) = 1. Note that for saturated probes beyond the maximum of the degredation hook d^N^(x) = 1 can be interpreted as the apparently absent 3′/5′-bias in the asymptotic range of hybridization.

The biased probe intensities are corrected according to

(27) $${\text{I}}_{p}^{\text{P,x-corr}}{\text{=I}}_{p}^{p}\text{/C}\left(\text{x,y}\right)$$

Hence, each probe intensity is rescaled according to its position (x = k or L) and the hybridization mode of its probe set according to y. Consequently, probe intensities taken from the non-specific hybridization range remain uncorrected. With increasing degree of specific hybridiztaion the probes are progressively scaled up with increasing distance from the 3′-end of the transcript. The maximum correction applies to probe sets in the S-hybridzation range. MM probe intensities are scaled using the mean logged MM-intensity of the probe set as argument. In the supplementary material ( Additional file 1) we compare and discuss both options of using relative and absolute probe positions.

The 3′/5′ correction algorithm is implemented in R and builds upon the popular affy package [16]. The respective program and data are available under http://www.izbi.uni-leipzig.de/downloads_links/programs/rna_integrity.php and as Bioconductor package *AffyRNADegradation*.

## Competing interests

Both authors declare that they have no competing interests.

## Authors’ contributions

HB and MF: Conceived and designed this study, performed data analysis; HB: developed theory and wrote large parts of the manuscript. MF: Wrote the programs and performed the calculations. Both authors read and approved the final manuscript.

## Supplementary Material

Additional file 1 Supplementary text addressing the probe positional characteristics of different gene chip types; the apparent degradation index for combined specific and non-specific probe signals and the correction of the 3′/5′ bias of pobe intensities and gene expression values (L-versus-k positional scaling). Click here for file

## References

[B1] Lee J Hever A Willhite D Zlotnik A Hevezi P Effects of RNA degradation on gene expression analysis of human postmortem tissues FASEB J 2005 04 3552fje 10.1096/fj.04-3552fje 15955843

[B2] Copois V Bibeau F Bascoul-Mollevi C Salvetat N Chalbos P Bareil C Candeil L Fraslon C Conseiller E Granci V Mazière P Kramar A Ychou M Pau B Martineau P Molina F Rio MD Impact of RNA degradation on gene expression profiles: Assessment of different methods to reliably determine RNA quality J Biotechnol 2007 127 549 559 10.1016/j.jbiotec.2006.07.032 16945445

[B3] Dumur CI Nasim S Best AM Archer KJ Ladd AC Mas VR Wilkinson DS Garrett CT Ferreira-Gonzalez A Evaluation of quality-control criteria for microarray gene expression analysis Clin Chem 2004 50 11 1994 2002 10.1373/clinchem.2004.033225 15364885

[B4] Popova T Mennerich D Weith A Quast K Effect of RNA quality on transcript intensity levels in microarray analysis of human post-mortem brain tissues BMC Genomics 2008 9 91 10.1186/1471-2164-9-91 18298816 PMC2268927

[B5] Fleige S Pfaffl MW RNA integrity and the effect on the real-time qRT-PCR performance Molecular Aspects of Medicine 2006 27 126 139 10.1016/j.mam.2005.12.003 16469371

[B6] Auer H Lyianarachchi S Newsom D Klisovic MI Marcucci G Kornacker K Chipping away at the chip bias: RNA degradation in microarray analysis Nat Genetics 2003 35 4 292 293 10.1038/ng1203-292 14647279

[B7] Viale A Li J Tiesman J Hester S Massimi A Griffin C Grills G Khitrov G Lilley K Knudtson K Ward B Kornacker K Chu C-Auer H Brooks AI Big results from small samples: evaluation of amplification protocols for gene expression profiling J Biomol Tech 2007 18 3 150 161 17595311 PMC2062549

[B8] Ma C Lyons-Weiler M Liang W LaFramboise W Gilbertson JR Becich MJ Monzon FA In vitro transcription amplification and labeling methods contribute to the variability of gene expression profiling with DNA microarrays J Mol Diagn 2006 8 2 183 192 10.2353/jmoldx.2006.050077 16645204 PMC1867595

[B9] Upton GJG Sanchez-Graillet O Rowsell J Arteaga-Salas JM Graham NS Stalteri MA Memon FN May ST Harrison AP On the causes of outliers in Affymetrix GeneChip data Briefings in Functional Genomics & Proteomics 2009 8 3 199 212 10.1093/bfgp/elp027 19734302

[B10] Thompson K Pine PS Rosenzweig B Turpaz Y Retief J Characterization of the effect of sample quality on high density oligonucleotide microarray data using progressively degraded rat liver RNA BMC Biotechnol 2007 7 1 57 10.1186/1472-6750-7-57 17854504 PMC2082023

[B11] Tomita H Vawter MP Walsh DM Evans SJ Choudary PV Li J Overman KM Atz ME Myers RM Jones EG Watson SJ Akil H William E Bunne J Effect of agonal and postmortem factors on gene expression profile: quality control in microarray analyses of postmortem human brain Biological Psychatry 2004 55 346 352 10.1016/j.biopsych.2003.10.013 PMC3098566 14960286

[B12] Weis S Llenos IC Dulay JR Elashoff M Martínez-Murillo F Miller CL Quality control for microarray analysis of human brain samples: the impact of postmortem factors, RNA characteristics, and histopathology Journal of Neuroscience Methods 2007 165 2 198 209 10.1016/j.jneumeth.2007.06.001 17628689

[B13] Schroeder A Mueller O Stocker S Salowsky R Leiber M Gassmann M Lightfoot S Menzel W Granzow M Ragg T The RIN: an RNA integrity number for assigning integrity values to RNA measurements BMC Mol Biol 2006 7 1 3 10.1186/1471-2199-7-3 16448564 PMC1413964

[B14] Spiess A-N Mueller N Ivell R Amplified RNA degradation in T7-amplification methods results in biased microarray hybridizations BMC Genomics 2003 4 1 44 10.1186/1471-2164-4-44 14606961 PMC280674

[B15] Wilson CL Miller CJ Simpleaffy: a bioconductor package for affymetrix quality control and data analysis Bioinformatics 2005 21 18 3683 3685 10.1093/bioinformatics/bti605 16076888

[B16] Gautier L Cope L Bolstad BM Irizarry RA affy–analysis of Affymetrix GeneChip data at the probe level Bioinformatics 2004 20 3 307 315 10.1093/bioinformatics/btg405 14960456

[B17] Held GA Grinstein G Tu Y Modeling of DNA microarray data by using physical properties of hybridization Proc Natl Acad Sci USA 2003 100 13 7575 7580 10.1073/pnas.0832500100 12808153 PMC164628

[B18] Burden CJ Pittelkow YE Wilson SR Statistical analysis of adsorption models for oligonucleotide microarrays Stat App in Gen and Mol Biol 2004 3 35 10.2202/1544-6115.1095 16646815

[B19] Binder H Kirsten T Loeffler M Stadler P The sensitivity of microarray oligonucleotide probes - variability and the effect of base composition J Phys Chem B 2004 108 46 18003 18014 10.1021/jp049593g

[B20] Binder H Thermodynamics of competitive surface adsorption on DNA microarrays J Phys Cond Mat 2006 18 18 S491 S523 10.1088/0953-8984/18/18/S02

[B21] Lee Y-S Chen C-H Tsai C-N Tsai C-L Chao A Wang T-H Microarray labeling extension values: laboratory signatures for Affymetrix GeneChips Nucl Acids Res 2009 37 8 e61 10.1093/nar/gkp168 19295132 PMC2677891

[B22] Van Gelder RN von Zastrow ME Yool A Dement WC Barchas JD Eberwine JH Amplified RNA synthesized from limited quantities of heterogeneous cDNA Proceedings Of The National Academy Of Sciences Of The United States Of America 1990 87 5 1663 1667 10.1073/pnas.87.5.1663 1689846 PMC53542

[B23] Garneau NL Wilusz J Wilusz CJ The highways and byways of mRNA decay Nat Rev Mol Cell Biol 2007 8 2 113 126 10.1038/nrm2104 17245413

[B24] Halperin A Buhot A Zhulina EB Sensitivity, specificity, and the hybridization isotherms of DNA Chips Biophys J 2004 86 2 718 730 10.1016/S0006-3495(04)74150-8 14747310 PMC1303922

[B25] Hekstra D Taussig AR Magnasco M Naef F Absolute mRNA concentrations from sequence-specific calibration of oligonucleotide arrays Nucl Acids Res 2003 31 7 1962 1968 10.1093/nar/gkg283 12655013 PMC152799

[B26] Burden C Pittlekow Y Wilson S Adsorption models of hybridisation and post-hybridisation behaviour on oligonucleotide microarrays J Phys Condens Matter 2006 18 5545 5565 10.1088/0953-8984/18/23/024

[B27] Binder H Preibisch S GeneChip microarrays - signal intensities, RNA concentrations and probe sequences J Phys Cond Mat 2006 18 S537 S566 10.1088/0953-8984/18/18/S04

[B28] Held GA Grinstein G Tu Y Relationship between gene expression and observed intensities in DNA microarrays—a modeling study Nucl Acids Res 2006 34 e70 10.1093/nar/gkl122 16723429 PMC1472623

[B29] Binder H Krohn K Burden C Washing scaling of microarray expression BMC Bioinforma 2010 11 291 10.1186/1471-2105-11-291 PMC2901370 20509934

[B30] Binder H Bruecker J Burden CJ Non-specific hybridization scaling of microarray expression estimates - a physico-chemical approach for chip-to-chip normalization J Phys Chem B 2009 113 2874 2895 10.1021/jp808118m 19708217

[B31] Group TTABPW Expression profiling — best practices for data generation and interpretation in clinical trials Nat Rev Genet 2004 5 3 229 237 14970825 10.1038/nrg1297

[B32] Raman T O’Connor T Hackett N Wang W Harvey B-G Attiyeh M Dang D Teater M Crystal R Quality control in microarray assessment of gene expression in human airway epithelium BMC Genomics 2009 10 1 493 10.1186/1471-2164-10-493 19852842 PMC2774870

[B33] Binder H Preibisch S “Hook” calibration of GeneChip-microarrays: theory and algorithm Algorithms for Molecular Biology 2008 3 12 10.1186/1748-7188-3-12 18759985 PMC2546411

[B34] Binder H Krohn K Preibisch S “Hook” calibration of GeneChip-microarrays: chip characteristics and expression measures Algorithms for Molecular Biology 2008 3 11 10.1186/1748-7188-3-11 18759984 PMC2543012

[B35] Salisbury J Hutchison KW Wigglesworth K Eppig JJ Graber JH Probe-level analysis of expression microarrays characterizes isoform-specific degradation during mouse oocyte maturation PLoS One 2009 4 10 e7479 10.1371/journal.pone.0007479 19834616 PMC2759528

[B36] Stalteri M Harrison A Interpretation of multiple probe sets mapping to the same gene in Affymetrix GeneChips BMC Bioinformatics 2007 8 1 13 10.1186/1471-2105-8-13 17224057 PMC1784106

[B37] Burden C Binder H Physico-chemical modelling of target depletion during hybridisation on oligonulceotide microarrays Phys Biol 2010 7 016004 20026877 10.1088/1478-3975/7/1/016004

[B38] Opitz L Salinas-Riester G Grade M Jung K Jo P Emons G Ghadimi B Beissbarth T Gaedcke J Impact of RNA degradation on gene expression profiling BMC Medical Genomics 2010 3 1 36 10.1186/1755-8794-3-36 20696062 PMC2927474

[B39] Archer KJ Guennel T An application for assessing quality of RNA hybridized to Affymetrix GeneChips Bioinformatics 2006 22 21 2699 2701 10.1093/bioinformatics/btl459 16935927

[B40] Archer KJ Dumur CI Joel SE Ramakrishnan V Assessing quality of hybridized RNA in affymetrix GeneChip experiments using mixed-effects models Biostat 2006 7 2 198 212 10.1093/biostatistics/kxj001 16135694

[B41] Binder H Preibisch S Berger H Grützmann R, Pilarski C Calibration of microarray gene-expression data Methods in Molecular Biology 2009 575 New York: Humana Press 376 407 10.1007/978-1-59745-545-9_20 19882273

