# Supplementary material for: Estimating RNA-quality using GeneChip microarrays
Source: BMC Genomics. 2012 May 14;13:186. doi: 10.1186/1471-2164-13-186 (PMC3519671; doi:10.1186/1471-2164-13-186)
Supplement: Additional file 1 — Supplementary text addressing the probe positional characteristics of different gene chip types; the apparent degradation index for combined specific and non-specific probe signals and the correction of the 3′/5′ bias of pobe intensities and gene expression values (L-versus-k positional scaling). [file 1471-2164-13-186-S1.pdf]

Supplementary text

Estimating RNA-quality using GeneChip microarrays

Mario Fasold[1,2] and Hans Binder[1,2]*

[1] Interdisciplinary Center for Bioinformatics; Universität Leipzig, D-4107 Leipzig, Haertelstr. 16-18
[2] LIFE
[3] UfZ

* Corresponding author: E-mail: binder@izbi.uni-leipzig.de, fax: ++49-341-9716679

Table of contents

1. Probe positional characteristics of different GeneChip types 2

2. Positional distribution of specific and non-specific hybridization of HG-U133 plus2 chips 3

3. Apparent degradation index for combined specific and non-specific probe signals 4

4. Correcting the 3'/5' bias of pobe intensities and gene expression values: L-versus-k positional scaling 7

5. References 10

1. Probe positional characteristics of different GeneChip types

Figure S 1 provides an overview over selected probe design characteristics of different GenChip types. Probes have been aligned to their target transcripts using consensus and exemplar sequences provided by Affymetrix. It shows that the mean position of the first and of the last probe in the probe sets can strongly vary between the different chip types giving rise to a wide range of <ΔL>-values. These differences refer in first instance to arrays of older and newer generations (e.g., the human genome HGU95a and HG133a arrays and the mouse genome MG74a and MOE430a arrays, respectively). On the other hand, the average span covered by the probe sets is relatively constant for all chip types considered.

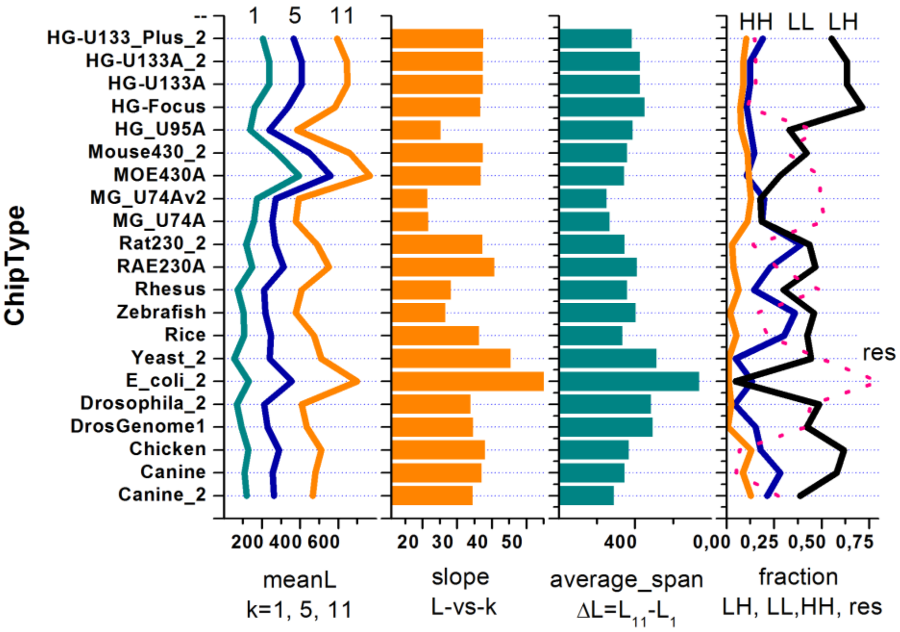

Figure S 1: Probe and probe set characteristics for different GeneChip microarrays: the mean positions of the 1th (nearest to the 3' end of the transcript), 5th and 11th (nearest to the 5' end) probe averaged over all probe sets; the slope of the linear regression of the mean probe position L versus the respective probe index for all probes (<ΔL>); the average transcript range covered by the probe sets (average span) and the fraction of probe sets from the LH, LL and HH ranges and the residual fraction not contained in one of the three ranges (see the main paper for definitions).

2. Positional distribution of specific and non-specific hybridization of HG-U133 plus2 chips

We determined the mean positional distribution of specific and non-specific hybridization of human genome HG U-133 plus2 arrays using the same analysis as described in the main paper. The dominance of non-specific hybridization for probe sets near the 3'-end of the transcripts is similar for rat genome and human genome arrays showing that the effect is not limited to a particular GeneChip expression array type.

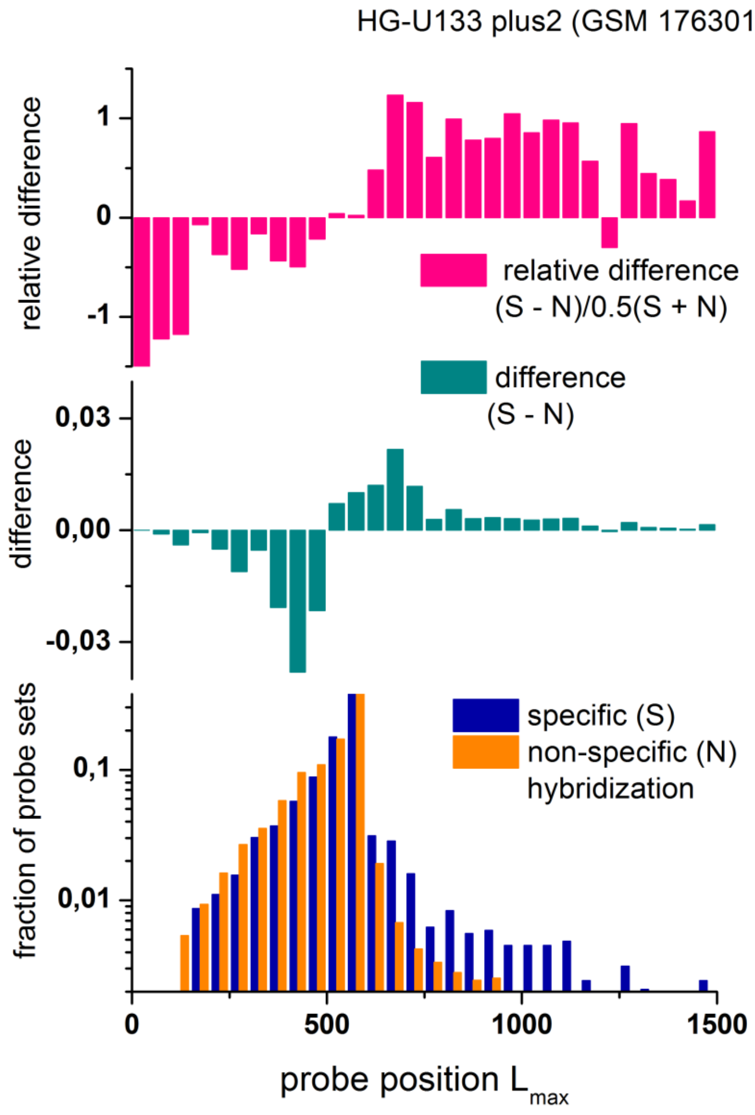

Figure S 2: Distribution of probe sets hybridized predominantly with specific and non-specific transcripts as a function of the position of the last probe of each probe set. The distributions are calculated using an example taken from the tissue data set.

3. Apparent degradation index for combined specific and non-specific probe signals

Let us consider the linear range of hybridization. In this special case Eq. (6) in the main paper transforms into a linear function of the true ratio $r_{3'/5'}$,

$$r^{app}_{5'/3'} = A \cdot r_{5'/3'} + B \qquad . \qquad (1)$$

We further assume that the apparent degradation ratio is calculated as mean value averaged over all probe sets of the microarray. The slope A and intercept B of the linear function are,

$$A = \frac{\langle r_x \rangle}{1 + a_{3'}} \quad \text{and} \quad B = \frac{a_{5'}}{1 + a_{3'}}$$

$$\text{with} \quad \langle r_x \rangle = \frac{\langle K^{P,h}_{5'} \cdot w^{P,h}_{5'} \rangle}{\langle K^{P,h}_{3'} \cdot w^{P,h}_{3'} \rangle} \quad , \qquad . \qquad (2)$$

$$a_p \equiv \frac{\langle d \cdot x^N_p \rangle}{\langle d_{3'} \cdot x^S_{3'} \rangle} \approx \frac{\%N}{1 - \%N} \cdot b_p \quad \text{and} \quad b_p = \frac{d \cdot N_{chip}}{\langle S_{3'} \rangle_S} \cdot \frac{\langle K^{P,N}_p \cdot w^{P,N}_p \rangle}{\langle K^{PM,S}_{3'} \cdot w^{P,S}_{3'} \rangle}$$

Particularly, $K_p^{P,N} \cdot w_p^{P,N}$ and thus r_x and a_p are functions of the probe sequence of the involved probes and $[S]_{3'}$ is the real concentration of the specific transcripts interrogated by the 3'-probes. The angular brackets denote averaging over all relevant probe sets. They are hybridized to different amounts by specific and non-specific transcripts in a competitive fashion. We assume a simple two state approximation classifying the probe sets either as completely 'present' or as completely 'absent', i.e. hybridized exclusively by the former or latter transcripts, respectively. Their fractions among all probe sets are given by %N and 1-%N, respectively.

Averaging the binding and washing constants over a sufficient large number of probes virtually cancels out the probe specificity yielding $a \equiv \langle a_{5'} \rangle \approx \langle a_{3'} \rangle$ and $\langle r^S_x \rangle \approx 1$ which transforms Eq. (1) into the simple form

$$r^{app}_{5'/3'} = \frac{r_{5'/3'} + a}{1 + a} \qquad . \qquad (3)$$

The slope and intercept of the $r_{5'/3'}^{app}$ – versus - $r_{5'/3'}$ line,

$$\text{slope} = \frac{1}{1 + a} \propto \frac{1 - \%N}{1 + \%N \cdot (b - 1)} \quad \text{and} \quad \text{intercept} = \text{slope} \cdot a = \frac{\%N \cdot b}{1 + \%N \cdot (b - 1)} \quad , \qquad (4)$$

depend both on the fraction of absent probes (see also Eq. (2)). Eq. (4) predicts that the slope and thus the sensitivity of the apparent degradation index decreases with increasing fraction of absent probe sets (%N). Insertion of Eq. (4) into Eq. (3) provides the apparent ratio as a function of %N,

$$r^{app}_{5'/3'} = \frac{\%N \cdot \; b - r_{5'/3'} \; + r_{5'/3'}}{\%N \cdot (b - 1) + 1} \qquad . \qquad (5)$$

The plot of $r_{5'/3'}^{app}$ – versus - $r_{5'/3'}$ data provides a linear function the slope and intercept of which depend on the fraction of absent probes. Also the plot of $r_{5'/3'}^{app}$ – versus - %N provides a linear function the slope and intercept of which depend on the true degradation ratio. An example of both cases is shown in Figure S 3 using the human body index data set.

Panel a of Figure S 3 compares the d^k 5'/3'-intensity ratios of the 677 array hybridizations of the human tissue data set calculated as average value over all probe sets of each array or, alternatively, over the sub-ensembles of predominantly specifically (S) and non-specifically (N) hybridized probe sets in terms of a rank plot. The obtained end mean ratios of the N-probe sets are virtually invariant among all arrays whereas that of the S-probes markedly vary, as expected. The change of the d^k parameters of all probes takes an intermediate position. The slope of the respective curve is roughly only half as large as that of the S-probes (see red bars in Figure S 3a). This result is intuitively

plausible because in this experiment about 50% of all probe sets of the arrays are absent, i.e. non-specifically hybridized, and thus they do not contribute to the 5'/3'-bias (see Figure S 3c).

A more detailed evaluation of the functional relation between the 'true' degradation index estimated in the S-hybridization regime and the apparent one obtained from all probe sets is given in the methodical part below. It predicts a linear dependence between the apparent and the true degradation ratios where the slope is expected to decrease with increasing %N (see Eqs. (3) and (4) in the methodical part). The data of the tissue data set confirm this prediction (Figure S 3b). Note that the obtained slope estimates the sensitivity of the apparent 5'/3'-ratio to determine the true 5'/3'-bias. The data show that the mean sensitivity of estimating the degradation level using all probes (slope=0.15...0.33) is less than one third compared with the measure based on the specific probes (slope=1). Theory also predicts that the apparent 5'/3'-ratio directly varies with the fraction of specifically hybridized probe sets, (see Eq. (5)). The insertion in Figure S 3b confirms this prediction for the tissue data set.

Hence, the apparent degradation ratio derived from the simple affy-slope intensity measures is strongly modulated by the fraction of non-specifically hybridized 'absent' probes leading potentially to the systematic overestimation of RNA quality. Contrarily, the proposed use of specifically hybridized probes largely removes this bias from the data and provides a reliable measure of the degradation degree which can be consistently compared between different arrays.

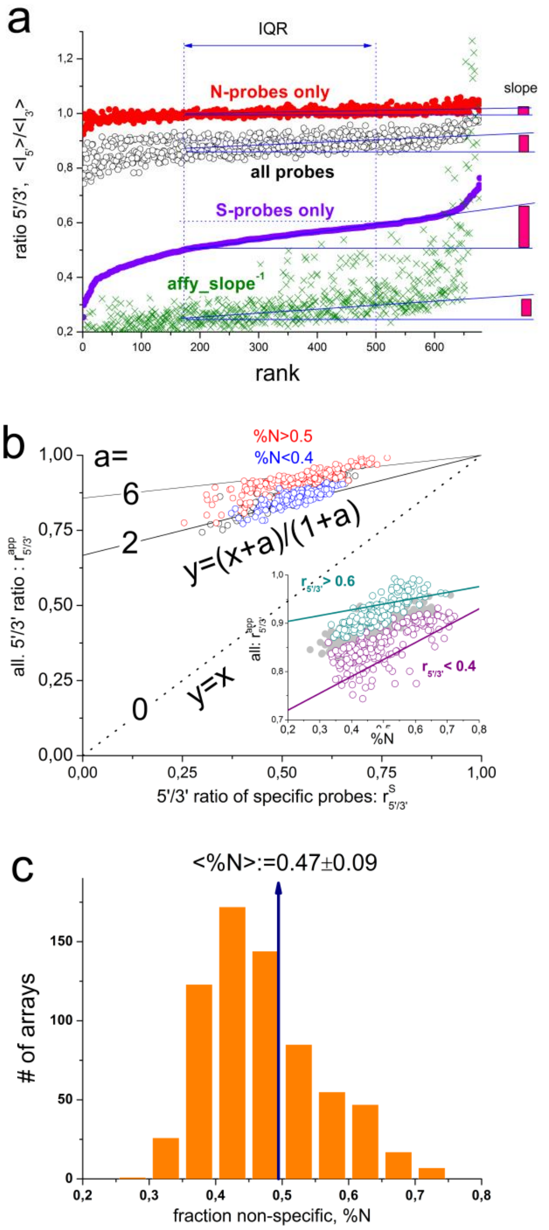

Figure S 3**:** The effect of non-specific hybridization on the apparent degradation level: Panel a shows the 5'/3'-intensity ratios of different subensembles of probes (all, only non-specifically hybridized and only specifically hybridized probe sets) taken from the arrays of the human tissue data set. The data were ranked according to the 5'/3'-ratios of the specific subsets. The 5'- and 3'-values refer to probes with indices k=11 and 1 of sets containing 11 probes. 'affy_slope' denotes the slope of the degradation plot as calculated by the bioconductor package 'affy'. The red bars indicate the increment of the degradation ratio within the interquartile range (IQR) of the different data ensembles. Panel b: Correlation plot between the 'apparent' 5'/3'-intensity ratios of all probes and the 'true' ratios as reported by the specifically hybridized probes. The lines are calculated using the theoretical model (see Eq. (3) and text). Red and blue symbols refer to arrays with large and small fractions of absent probes, %N, respectively. The insertion shows the correlation plot between $r_{5'/3'all}$ and %N. The different colors refer to large and small values of $r_{5'/3'}$, respectively. The lines are calculated using Eq. (5). Panel c shows the distribution of absent probe sets of the human tissue data set. %N was calculated using the hook-method [1-2]. <%N> is the mean over all arrays studied and '±' the standard deviation.

4. Correcting the 3'/5' bias of pobe intensities and gene expression values: L-versus-k positional scaling

To discuss potential differences of the obtained expression values after index- and positional-based corrections we assume predominant specifically hybridized probes. This special case also implies that expression and intensity values roughly agree owing to the small effect of non-specific hybridization. We also assume an exponentially decaying correction function for sake of simplicity ($d_{\infty}^{x}=0$ in Eq.10 in the main paper). The logged mean intensity averaged over a selected probe set then becomes after correction (use Eq. **Fehler! Verweisquelle konnte nicht gefunden werden.** with $f^{S}=1$)

$$\left\langle \log I_p^{P,x-corr} \right\rangle_{pset} = \left\langle \log I_p^{P} \right\rangle_{pset} + \left\langle x \right\rangle_{pset} / \lambda_x \cdot \ln 10 \quad . \qquad (6)$$

Importantly, the probe set averaged mean index is identical with the array-related mean index averaged over all probe sets of the array if all probe sets contain the same number of probes, i.e. $<k>_{pset} = <k>_{array}$. The equation applies to GeneSet arrays to a good approximation because the overwhelming majority of probe sets contains the same number of probes per set (usually $k_{max}=11$ and thus $<k>_{array}=5.5$). The index-correction (Eq. (6)) consequently scales all expression values referring to specific hybridization of one array by the same factor, or in log-scale, adds the same increment term ~ $\left\langle k \right\rangle_{array} / \lambda_k$. The correction is scaled solely by the degree of specific binding $f^{S}(y)$.

Contrarily, the positional correction applies a specific correction ~ $\left\langle L \right\rangle_{pset} / \lambda_L$ to each probe set. Note that the mean position of the probes of each probe set varies from set to set and thus it usually deviates from the mean value averaged over all probe sets on the array, i.e. $<L>_{pset} \neq <L>_{array}$.

Making use of the $<\Delta L>$ defined in the main paper (Eq. 1) then allows to link the index- and position-corrected mean intensities:

$$\left\langle \log I_p^{P,L-corr} \right\rangle_{pset} \approx \left\langle \log I_p^{P,k-corr} \right\rangle_{pset} + \left(\frac{\left\langle L \right\rangle_{pset}}{\left\langle L \right\rangle_{array}} - 1 \right) \cdot \left\langle k \right\rangle_{array} / \lambda_k \cdot \ln 10 \quad . \qquad (7)$$

Eq. (7) shows that both corrections agree if the set averaged mean position of the probes agrees with the respective total array average ($\left\langle L \right\rangle_{pset} = \left\langle L \right\rangle_{array}$). For probe sets with a mean position nearer the 3'-transcript end (i.e. $<L>_{pset} < <L>_{array}$) the index-based correction exceeds that of the position-based correction whereas for $<L>_{pset} < <L>_{array}$ this relation reverses. The analysis of the series of different array types considered shows that the 25%- and 75%-percentiles of the distributions of $<L>_{pset}$ provide correction factors $\left\langle L \right\rangle_{pset} / \left\langle L \right\rangle_{array} - 1$ = -0.4 – -0.5 and +0.4 - +0.5, respectively (see Figure S 1). Hence, the position-specific correction deviates from the index-based correction by more or less than +0.1/-0.1 for 50% of the probe sets if one assumes $\lambda_k=10$ which refers to relatively strong degradation (e.g. RIN=6.1 of the ratQC data set, see

Figure S 4b). In the mix-range one expects the same qualitative relations between both options for correcting the 3'-bias of expression values the amplitude of which is however systematically reduced due to the down-weighting of the effect ($f^{S}<1$).

Hence, the index correction effectively applies the same factor to all probe sets which is scaled solely by the degree of specific hybridization. Contrarily, the positional correction applies a specific factor to each probe-set. The M/A-plot of the L-corrected – versus- k-corrected intensities shows that the probe sets located on the average nearer to 3'-end of the transcript are corrected to a less degree (see red symbols in

Figure S 4) than probe sets located more distant from the 3'-transcript end (see green symbols). The respective expression data shown in panel c – f of Figure S 4 show the same general trend as discussed for the intensity data.

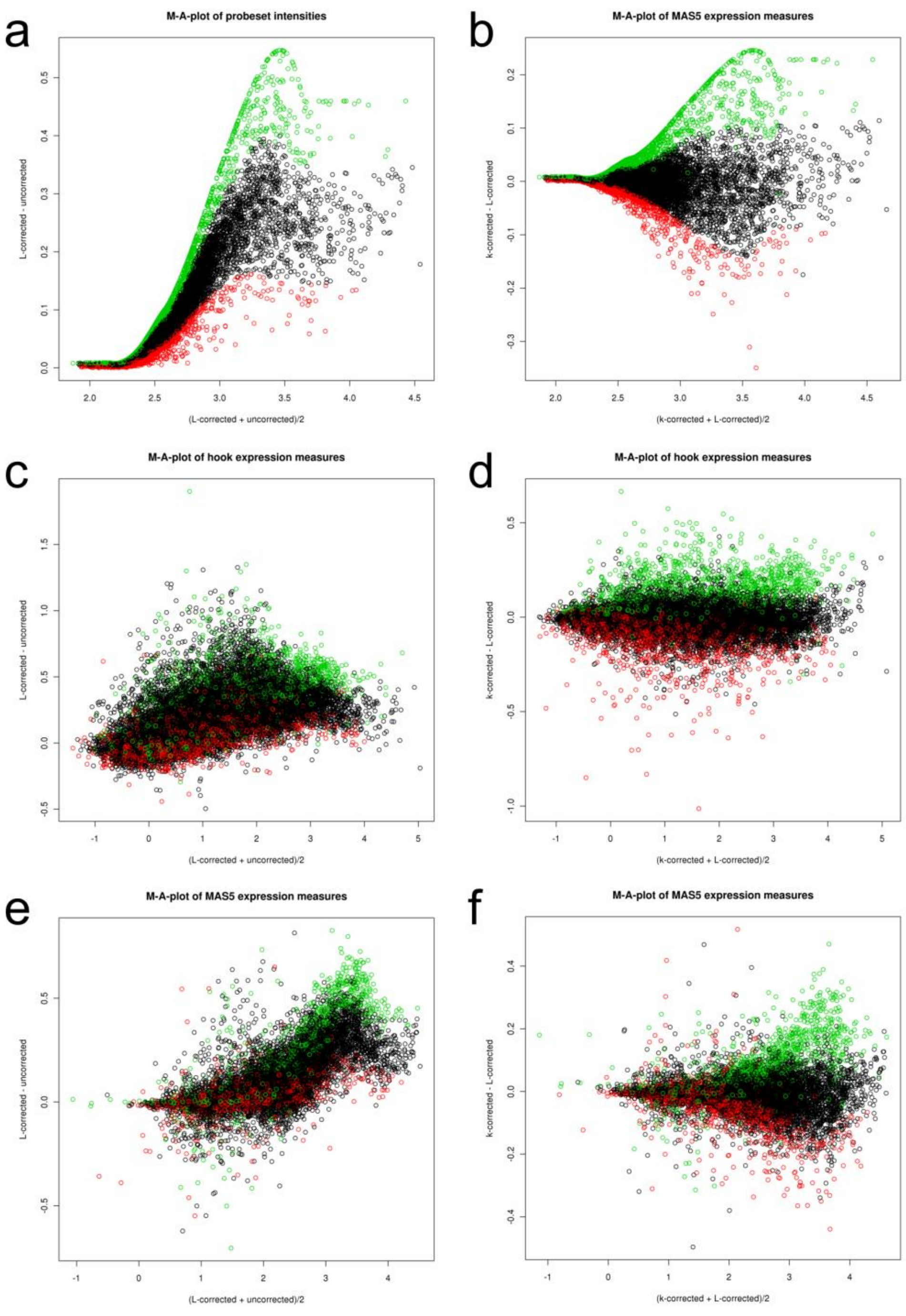

Figure S 4: M-A plots of the L-corrected – versus – uncorrected intensities (panel a), and L-corrected-versus- k-corrected intensities (b) of the RIN=6.1 sample of the RatQC series. Each symbol refers to the logged mean of the probe intensities averaged over the probe set. Panel c – f show the respective M-A plots of expression values obtained after intensity calibration using the hook method (panel c and d) and MAS5 (e and f). The lower quartile of probe sets which are located closer to the 3'-end of the transcripts are coloured in red and the upper quartile of probe sets located far away from the 3'-end are colored in green.

Panel a and b of Figure S 5 show the tongs-plot of a strongly degraded array which is taken from the rat-QC data set (RIN=6.1) before and after correction. It demonstrates that the tongs-opening is largely removed from the intensity data after correction. In Figure S 5c we compare the frequency distributions of expression values obtained after hook-calibration of uncorrected, index- and position-based 3'-corrected intensity data. The correction shifts the right flank towards larger expression

values. Both correction-methods affect the distribution nearly identically. The distribution reflects the mean correction amplitude without emphasis on the individual probe sets.

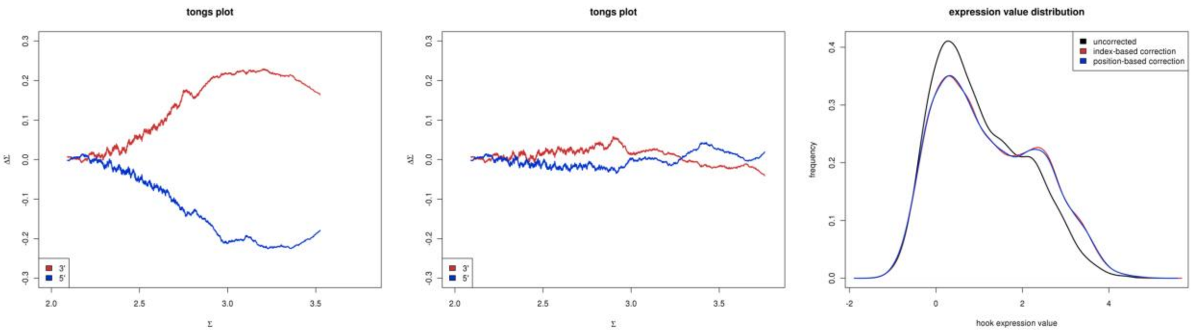

Figure S 5: Tongs plot for an array hybridized with strongly degraded RNA in the RatQC experiment (RIN=6.1) before and after correction of the probe intensities for the 3’-bias. The right panel shows the respective distributions of expression values before correction and after index- and position-based correction. The right flank clearly shifts towards larger expression values after correction.

5. References

1. Binder H, Preibisch S: **"Hook" calibration of GeneChip-microarrays: Theory and algorithm**. *Algorithms for Molecular Biology* 2008, **3:12**.
2. Binder H, Krohn K, Preibisch S: **"Hook" calibration of GeneChip-microarrays: chip characteristics and expression measures**. *Algorithms for Molecular Biology* 2008, **3:11**.
